# Understanding the spatial determinants of the Oxford Classic prognostic signature for high-grade serous ovarian cancer

**DOI:** 10.1038/s43856-026-01708-1

**Published:** 2026-06-06

**Authors:** Alexandru Stihi, Christopher Yau

**Affiliations:** 1 Nuffield Department for Women’s and Reproductive Health, University of Oxford, Oxford, UK; 2 Health Data Research UK, London, UK

**Keywords:** Computational biology and bioinformatics, Cancer

## Abstract

**Background:**

The Oxford Classic (OxC) prognostic signature classifies high-grade serous ovarian cancer (HGSOC) into five transcriptional programs, with epithelial-to-mesenchymal transition (EMT) marking poor prognosis. While successful in bulk transcriptomics, the spatial organisation of these programs within the tumour microenvironment remains unexplored.

**Methods:**

We developed the Signature-guided Zero-inflated Beta Variational Autoencoder (Sig-ZIB-VAE), a deep learning deconvolution method tailored for spatial transcriptomics data, and applied it to a large-scale HGSOC cohort comprising 94 tumours to quantify spatial cellular organisation. Prognostic significance was assessed using penalised Cox proportional hazards regression integrating clinical, molecular, and spatial features.

**Results:**

Here we show that EMT cells form dense homotypic clusters broadly depleted from stromal and immune neighbourhoods, yet maintain selective monocyte co-localisation at cluster boundaries. EMT-high tumours display enhanced spatial reorganisation characterised by increased clustering and connectivity, forming locally concentrated mesenchymal-rich domains. Survival analysis confirms EMT-high status as an adverse prognostic factor.

**Conclusions:**

Critically, spatial metrics of immune cell organisation—particularly monocyte connectivity and clustering—provide substantially stronger prognostic discrimination than EMT proportion alone, demonstrating that tumour microenvironment architecture supersedes cellular composition in determining clinical outcomes in HGSOC.

## Plain Language Summary

Ovarian cancer is often found late and can be hard to treat. The Oxford Classic is a test that looks at patterns of gene activity in cancer cells and can identify a more aggressive type of tumour cell linked to poorer survival. Until now, it was unclear whether how these tumour cells are arranged within the tumour also affects patient outcomes.In this study, we applied the Oxford Classic to tumour samples while also analysing where different cancer and immune cells are located. We found that tumours with more aggressive cancer cells were linked to worse survival, confirming that the Oxford Classic works in this new setting. Importantly, we also showed that how immune cells are physically arranged within the tumour gives extra information about patient outcomes, beyond simply counting cell types. This suggests that the spatial organisation of cells in ovarian tumours plays an important role in determining how the disease progresses.

## Abstract (web summary)

Stihi et al., develop a deep learning framework for spatial transcriptomics data to investigate the spatial distribution of EMT programs in ovarian cancer. The results show that spatial organisation refines EMT-based stratification and provides stronger prognostic discrimination than cellular composition alone.

## Introduction

High-grade serous ovarian cancer (HGSOC) is the most common and aggressive subtype of epithelial ovarian cancers, accounting for the majority of ovarian cancer-related deaths worldwide^1^. Recent evidence indicates that one site of origin for HGSOC is the secretory epithelium of the distal fallopian tube^2^. This insight has motivated application of deep molecular analysis to examine the biology of fallopian tube epithelial (FTE) cells and precursor lesions to better capture the cellular programs underpinning HGSOC^3–5^.

The Oxford Classic (OxC) prognostic signature for HGSOC is based on a 52-gene panel^6^ that was derived from single-cell expression profiling of normal FTE cells. OxC associates tumours with one of five transcriptional programs: C3 (differentiated), C4 (KRT17 subtype), C10 (cell cycle), ciliated, and epithelial-to-mesenchymal transition (EMT). Among these, the EMT program has emerged as a marker of particularly poor prognosis in HGSOC, reflecting its role in invasion, metastasis, and immune evasion^7,8^. OxC has previously been successfully applied to bulk tumour transcriptomes, where deconvolution methods estimate the relative contributions of these FTE-derived programs to each tumour. However, while this approach captures overall abundance of programs such as EMT, it ignores the spatial dimension — potentially overlooking critical aspects of tumour heterogeneity, architecture, and microenvironmental interactions.

Recently, Yeh et al.^9^ used in situ imaging with high-plex RNA detection at single-cell resolution^10^ to map the spatial organisation of high-grade serous ovarian cancer and profiled more than 2.5 million cells in 130 tumours from 94 patients using the CosMx system. This included a discovery subset in which single-molecule imaging was applied to formalin-fixed and paraffin-embedded (FFPE) tissue microarrays (TMAs) and the expression of 960 genes was measured across 491,792 cells from 94 of the tumours. We sought to utilise this resource to investigate the existence of any spatial determinants of the Oxford Classic signature.

In this work, we translate the OxC prognostic signature to the 960-gene set on the CosMx spatial assay to investigate whether OxC programs, and EMT in particular, exhibit non-random spatial distributions within the tumour microenvironment of HGSOC. We develop a deep learning-based deconvolution method tailored for spatial transcriptomics data and integrate OxC-derived signatures with CosMx profiles to characterise the spatial organisation of EMT and other FTE programs across tumour tissue. Through this approach, we test the hypothesis that the OxC classification, initially designed for bulk transcriptomics, also encodes meaningful spatial information when applied to high-resolution tissue data. We show that EMT cells form spatially clustered, mesenchymal-rich domains that are broadly depleted from stromal and immune neighbourhoods, while maintaining selective co-localisation with monocytes at cluster boundaries. Survival analysis confirms that EMT-high status is associated with poor prognosis and demonstrates that spatial organisation provides stronger prognostic discrimination than EMT proportion alone. These findings indicate that the OxC classification encodes spatially meaningful biological organisation and that tumour microenvironment architecture is a key determinant of clinical outcomes in HGSOC.

## Methods

### Analytical overview

**Fig. 1:**
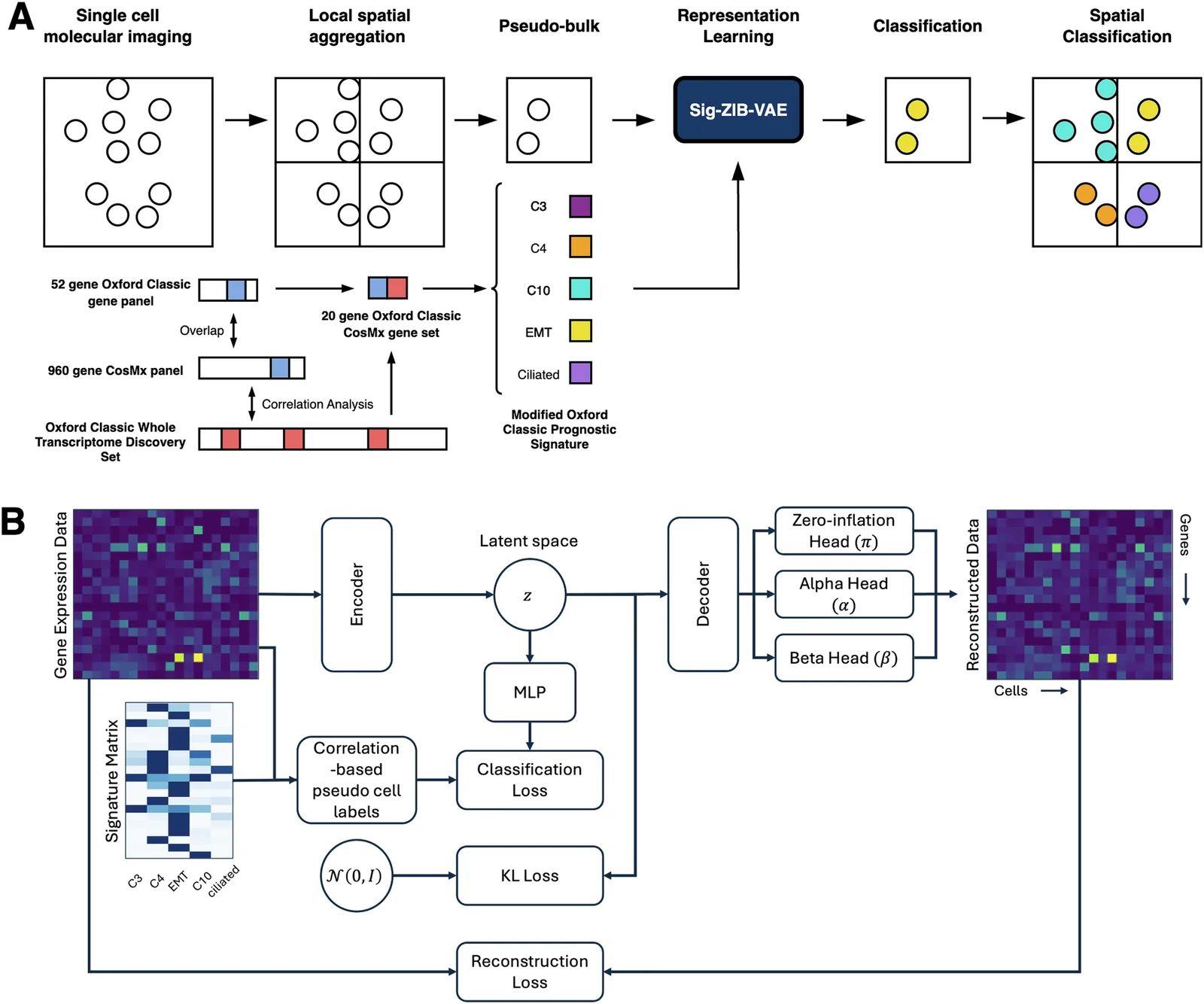
Sig-ZIB-VAE Methods Overview. **A** Analysis pipeline. Single-cell CosMx expression profiles are locally aggregated on a fixed grid to generate spatial tiles (pseudo-bulk) and restricted to a modified 20-gene Oxford Classic (OxC) panel obtained by intersecting the original 52-gene OxC set with the 960-gene CosMx discovery panel (Methods). Tile-level profiles are input to a signature-guided zero-inflated Beta variational autoencoder (Sig-ZIB-VAE) to learn low-dimensional representations and assign OxC programme probabilities (C3, C4, C10, EMT, ciliated). Predicted programme labels are mapped back to native cell coordinates to produce spatial program maps, from which cell interaction graphs are constructed and multi-scale spatial metrics are quantified. **B** Schematic overview of the Signature-guided Zero-Inflated Beta Variational Autoencoder (Sig-ZIB-VAE). The model encodes normalized gene expression data **X** into a latent representation **z** using an encoder network. Latent variables are sampled via the reparameterization trick and decoded through a shared trunk with three output heads estimating the zero-inflation probability (*π*) and Beta distribution parameters (*α*, *β*), which are used for data reconstruction. In parallel, **z** is passed through a classifier predicting cell-type probabilities, supervised by pseudo-labels derived from correlations with a reference signature matrix **S**. Training minimizes a composite loss combining reconstruction, Kullback–Leibler divergence, and classification terms.

To investigate the spatial organisation of OxC cell states in ovarian cancer, we developed a modified 20-gene signature panel by identifying the overlap between the original 52-gene OxC panel and the 960-gene CosMx spatial assay. Marker genes for the five FTE malignant subtypes (C3, C4, C10, ciliated, and EMT) were selected using the criteria described in Hu et al.^6^, with a relaxed dispersion threshold compared to the original OxC framework, thus maximising gene overlap while maintaining signature fidelity. The resulting gene set was used to construct a signature matrix, capturing the average expression profile of each cell state. The analytical pipeline used in this work proceeded through several stages (see Fig. 1A): starting from single-cell spatial transcriptomics data, we applied local spatial aggregation using a fixed grid to generate pseudo-bulk samples. These aggregated expression profiles were then normalized to the [0, 1] interval and analyzed using a Signature-guided Zero-inflated Beta Variational Autoencoder (Sig-ZIB-VAE) framework.

The Sig-ZIB-VAE architecture (Fig. 1B) explicitly models the bimodal, zero-inflated characteristics of the spatially-aggregated data through a multi-task learning framework. As such, the encoder network maps normalized gene expression profiles to a continuous latent space via a variational framework, where latent representations are sampled using the reparametrisation trick. The decoder consists of a shared trunk, followed by three specialized output heads that parameterize a zero-inflated Beta distribution: one head predicts zero-inflation probabilities (*π*) via *sigmoid* activation, where two additional heads generate the shape parameters (*α* and *β*) of the Beta distribution through *Softplus* transformations. To incorporate biological priors, we implemented a multi-layer perceptron (MLP) classifier operating on the latent space that predicts cell-type probabilities based on correlation-derived pseudo-labels generated by comparing input profiles to the reference signature matrix. This model is trained by jointly optimising three objectives: (1) reconstruction fidelity, through zero-inflated Beta likelihood optimisation, (2) latent space regularisation via Kullback–Leibler (KL) divergence with a standard Gaussian prior, and (3) classification accuracy relative to signature-correlated labels. This integrated approach enables the model to learn biologically interpretable latent representations while efficiently processing large-scale spatial datasets and capturing continuous transitions between cell states.

Following deconvolution, spatial neighbourhood graphs were constructed to quantify the tissue architecture, representing cells as nodes and cell-cell interactions as edges. Thus, four complementary spatial metrics were used: neighbourhood enrichment (assessing non-random spatial associations via permutation testing), co-localization quotient (CLQ, measuring directional infiltration patterns), Ripley’s L function (evaluating clustering behaviour across spatial scales via area under the curve), and degree centrality (quantifying cluster connectivity within the tissue network). Finally, to assess the prognostic significance of spatial features, we integrated baseline clinical covariates (age, stage, neoadjuvant chemotherapy, BRCA1/2 status, and tumour mutational burden) with EMT cell proportions and top-performing spatial metrics into penalized Cox proportional hazards models using elastic net regularisation. Missing clinical data were addressed through multiple imputation by chained equations (MICE) with 20 imputed datasets, and model coefficients were pooled to provide robust survival predictions accounting for both model and imputation uncertainty.

### Signature matrix, dataset and preprocessing

The signature matrix was acquired following the procedure detailed in ref. ^6^. The marker genes for the five FTE subtypes were first selected using the following cut-off points: (1) log_2_ fold change ≥1.5, (2) false discovery rate (FDR) ≤0.01; (3) dispersion ≥0.025 that was measured by variable loadings in the principal component analysis of The Cancer Genome Atlas (TCGA) RNAseq data^11^; (4) the correlation with each other markers was less than 0.9 to avoid redundancy. It should be noted that the dispersion cut-off threshold was lowered here (compared to the 0.2 threshold used in ref. ^6^), to improve overlap between the CosMx panel and the OxC signature, yielding 20 shared genes instead of 11. The signature matrix was then computed using the BSEQ-sc method^12^ which measures the average expression levels of the marker genes in each cell subtype. As a benchmark, CIBERSORTx^13^ was used to perform deconvolution analysis, which used the aforementioned signature matrix and *pseudo-bulk* data generated averaging TPM-normalized expression values across all malignant cells from the same patient per gene. This analysis generated scores of five transcriptomic signatures in each *pseudo-bulk* tumour sample, which can be interpreted as the proportion of the corresponding cell state within the tumour (see Supplementary Section 1).

Spatial transcriptomics data were obtained from the Discovery dataset described in Yeh et al.^9,14^. This dataset includes expression profiles of 960 genes across 94 tumours. For the present study, only cells that passed the original quality control filters were retained, and low-confidence cells were excluded from downstream analyses. Consequently, the final dataset comprised 491,792 cells, which included 314,191 malignant cells, 28,676 T cells and natural killer (NK) cells, 16,373 B cells, 45,549 monocytes, 606 mast cells, 72,861 fibroblasts/stromal cells and 13,536 endothelial cells. For brevity, detailed descriptions of data acquisition and preprocessing are provided in Yeh et al.^9^ and are not repeated here. The overlapping gene set between the CosMx panel and the OxC signature included the following genes: APOA1, DCN, DPP4, ENG, IGFBP5, IGFBP6, IL1B, IL1RN, KRT23, LDLR, MGP, MYH11, PIGR, PTGS1, RAMP1, RGS5, S100A4, SPP1, TPM2, UBE2C.

Next, cell count data underwent a two-step normalization procedure to facilitate meaningful comparisons. First, to account for differential expression magnitudes across genes, we applied gene-specific normalization by calculating scaling factors as the reciprocal of the total counts for each gene across all cells. The expression values were then multiplied by these factors, effectively standardizing the contribution of each gene. Second, to ensure compatibility with the signature matrix, each cell’s gene expression profile was rescaled to the [0, 1] interval. This normalization strategy preserves the relative expression patterns while enabling direct comparison with the reference signatures used in the *pseudo-bulk* deconvolution analysis.

**Fig. 2:**
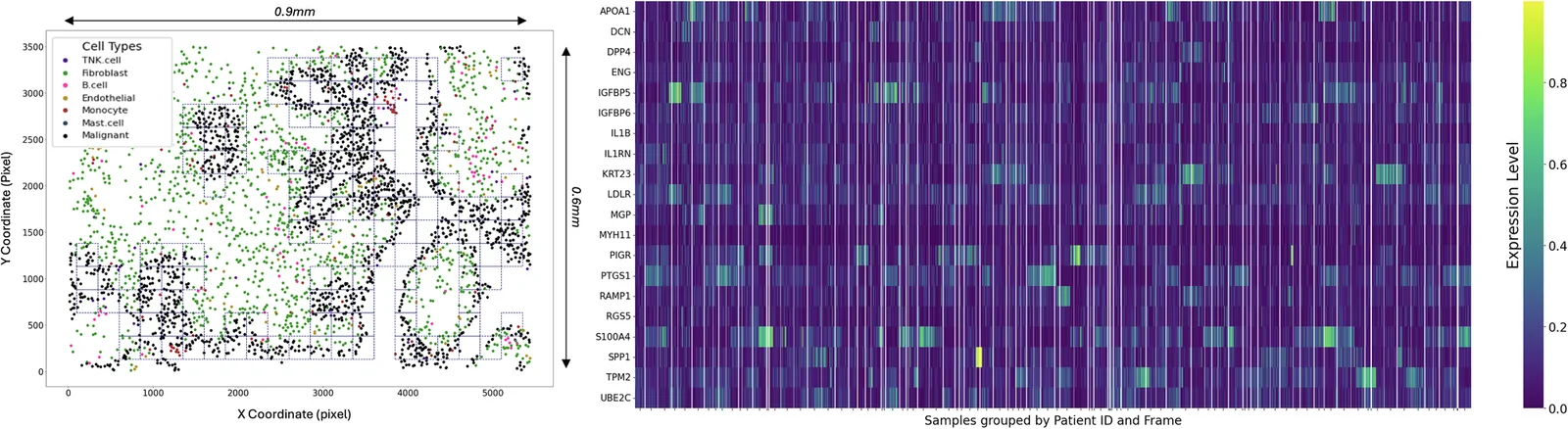
Spatial Expression Example. Left: Example FOV showing the spatial grid used for aggregating the expression data. Here, only the grid sections where malignant cells were detected are displayed. Right: Expression heatmap of selected genes across patient samples. The heatmap displays normalized expression levels of 20 genes(rows) across individual samples (columns) grouped by Patient ID and field of view (FOV). Expression values are normalized by total cell counts in every spatial grid cell. The white vertical lines are strictly used for delimitation purposes. Tick lines are demarcating boundaries between different patients, while thin lines are separating different FOVs from within the same patient.

Finally, to analyse spatial gene expression patterns, we implemented a grid-based spatial aggregation approach across malignant cells. This methodology enabled us to capture localized transcriptional signatures while maintaining adequate statistical power. As such, for each FOV (0.9 mm × 0.6 mm), a square grid with fixed dimensions (45 μm) was centered on the FOV to ensure optimal coverage (see Fig. 2). Within each grid cell, malignant cells were identified based on the cell designation established in ref. ^9^. To ensure statistical robustness, only grid cells containing at least 3 malignant cells were retained for downstream analysis. For each qualifying grid cell, we aggregated the normalized expression data for the 20 genes of interest and normalized by the total number of malignant cells in that specific grid. The resulting gene expression matrix is also provided in Figure 2. This approach allowed us to generate spatially resolved expression profiles that could be subjected to deconvolution analysis.

While the OxC framework successfully leveraged CIBERSORT^15^ to deconvolve bulk HGSOC data using single-cell signatures from FTE cells, several limitations inherent to traditional these deconvolution methods necessitate a more sophisticated approach. Statistical deconvolution methods such as CIBERSORT or CIBERSORTx^16^ rely on support vector regression with fixed reference matrices. This becomes computationally prohibitive as datasets scale to thousand of samples and cannot adapt to dataset-specific characteristics^17^. Additionally, these methods enforce discrete cell type assignments rather than capturing the continuous spectrum of cell states, limiting their ability to model biological processes like epithelial-mesenchymal transition that are critical in HGSOC^6^. In contrast, the methodology presented in this work addresses these limitation though a deep-learning based architecture that efficiently processes large-scale datasets, while its multi-task learning objective simultaneously optimizes gene expression reconstruction, latent representation learning, and cell-type classification. This integrated approach not only improves computational efficiency but also learns a continuous latent space that may capture gradual transitions between cell states—providing richer biological insights than traditional discrete deconvolution methods while maintaining interpretability through signature-guided learning.

Under University of Oxford institutional policy, no ethics review was required because this study involved only secondary analysis of previously published, fully de-identified data. The discovery dataset described in Yeh et al.^9^ were obtained from archival clinical FFPE tumor tissues, retrospectively procured from archival storage under an Institutional Review Board (IRB)-approved protocol (number 44615) and made publicly available^14^.

### Sig-ZIB-VAE description

Following the normalization procedure described previously, the resulting data demonstrated characteristics consistent with a zero-inflated beta distribution, with all values bounded within the [0, 1] interval. As such, inspired by the work of Lopez et al.^18,19^, this paper proposes the Signature-guided Zero-inflated Beta Variational Autoencoder (Sig-ZIB-VAE), a scalable method for tumour microenvironment deconvolution analysis, specifically developed for spatially aggregated gene expression data. This modeling procedure explicitly models the bimodal characteristics of the data, i.e., dropout events represented though zero-inflation and continuous values following a Beta distribution for expressed genes. Here, three objectives are jointly optimized in this multi-task framework: gene expression reconstruction, interpretable latent representation-guided though prior biological knowledge encoded in reference signatures, and cell-type classification. The mathematical formulation of the model is described below.

Let **S** = [**s**_1_, …, **s**_*K*_] ∈ [0, 1]^*P*×*K*^ denote a *P* × *K* gene expression signature matrix involving *P* genes and *K* cell type signatures. Let **X** = [**x**_1_, …, **x**_*N*_] ∈ [0, 1]^*P*×*N*^ denote a *P* × *N* normalized gene expression matrix involving *P* genes and *N* cells.

Then, for each cell *n*, we model the expression of gene *p* as a zero-inflated Beta distribution:

1 $${x}_{np} \sim \left\{\begin{array}{ll}0, \hfill & {\rm{with}}\,{\rm{probability}}{\pi }_{np} \hfill \\ {\rm{Beta}}({\alpha }_{np},{\beta }_{np}), & {\rm{with}}\,\,{\rm{probability}}1-{\pi }_{np}\end{array}\right.$$

where *π*_*np*_ ∈ [0, 1] is the zero-inflation probability, and *α*_*np*_, *β*_*np*_ > 0 are the shape parameters of the Beta distribution. The graphical representation of the proposed model can be seen in Fig. 1B. The encoder, denoted by *f*_encoder_ is a multi-layer neural network with parameters *θ*_*e*_ that maps the gene expression profile **x**_*n*_ ∈ [0, 1]^*P*^ to a latent representation:

2 $${{\bf{h}}}_{n}^{(e)}={f}_{{\rm{encoder}}}({{\bf{x}}}_{n};{\theta }_{e})$$

Then, the parameters of the latent distribution for each data point *n* are computed as linear transformations of the encoder hidden representations ${{\bf{h}}}_{n}^{(e)}$:

3a $${{\boldsymbol{\mu }}}_{n}={{\bf{W}}}_{\mu }{{\bf{h}}}_{n}^{(e)}+{{\bf{b}}}_{\mu }$$

3b $$\log {{\boldsymbol{\sigma }}}_{n}^{2}={{\bf{W}}}_{\sigma }{{\bf{h}}}_{n}^{(e)}+{{\bf{b}}}_{\sigma }$$

where ${{\boldsymbol{\mu }}}_{n}\in {{\mathbb{R}}}^{d}$ and ${{\boldsymbol{\sigma }}}_{n}^{2}\in {{\mathbb{R}}}^{d}$ denote the mean and variance of the approximate posterior distribution, ${{\bf{W}}}_{\mu },{{\bf{W}}}_{\sigma }\in {{\mathbb{R}}}^{d\times h}$ are learnable weight matrices, ${{\bf{b}}}_{\mu },{{\bf{b}}}_{\sigma }\in {{\mathbb{R}}}^{d}$ are bias terms. Here, *h* denotes the encoder hidden dimension, while *d* is the dimensionality of the latent space. The latent variable **z**_*n*_ is then sampled using the reparameterization trick^20^, which enables backpropagation through the stochastic sampling step:

4 $${{\bf{z}}}_{n}={{\boldsymbol{\mu }}}_{n}+{{\boldsymbol{\sigma }}}_{n}\odot {\boldsymbol{\epsilon }},\,{\boldsymbol{\epsilon }} \sim {\mathcal{N}}(0,{{\bf{I}}}_{d})$$

Here  ⊙ denotes element-wise multiplication and ***ϵ*** is a noise vector drawn from a standard *d*-dimensional Gaussian distribution.

Next, the decoder network maps the latent representation **z**_*n*_ to a lower-dimensional hidden representation:

5 $${{\bf{h}}}_{n}^{(d)}={f}_{{\rm{decoder}}}({{\bf{z}}}_{n};{\theta }_{d})$$

where *f*_decoder_ is a neural network with parameters *θ*_*d*_. The decoder consists of a shared trunk (common layers) followed by three specialized output heads, each responsible for producing the parameters of the zero-inflated Beta distribution:

6a $${{\boldsymbol{\pi }}}_{n}=\sigma \left({f}_{{\rm{zero}}}\right.({{\bf{h}}}_{n}^{(d)};{\theta }_{\pi })$$

6b $${{\boldsymbol{\alpha }}}_{n}={\rm{Softplus}}({f}_{\alpha }({{\bf{h}}}_{n}^{(d)};{\theta }_{\alpha }))+{\epsilon }_{\min }$$

6c $${{\boldsymbol{\beta }}}_{n}={\rm{Softplus}}({f}_{\beta }({{\bf{h}}}_{n}^{(d)};{\theta }_{\beta }))+{\epsilon }_{\min }$$

where**π**_*n*_ ∈ (0, 1) is the probability of observing a structural zero, **α**_*n*_, **β**_*n*_ > 0 are the shape parameters of the Beta distribution, *f*_zero_, *f*_*α*_, *f*_*β*_ are output layers (typically linear mappings) with learnable parameters *θ*_*π*_, *θ*_*α*_, *θ*_*β*_. *σ*( ⋅ ) denotes the sigmoid function, while the Softplus function ensures positivity and ${\epsilon }_{\min }$ is a small positive constant added for numerical stability. This structure allows the decoder to jointly capture the zero-inflation mechanism (*π*_*n*_) as well as the continuous variability in the data through the Beta distribution parameters (*α*_*n*_, *β*_*n*_). Finally, the reconstructed expression values are computed as the expected value of the zero-inflated Beta distribution, given below:

7 $${\mathbb{E}}[{x}_{np}]=(1-{\pi }_{np})\frac{{\alpha }_{np}}{{\alpha }_{np}+{\beta }_{np}}$$

The Sig-ZIB-VAE model requires three key inputs: (1) the aggregated gene expression profiles from the dataset, (2) a reference signature matrix **S** containing cell type-specific molecular signatures, and (3) cell type labels derived from a correlation-based preprocessing step.

The correlation-based procedure is an essential step that transforms continuous expression data into discrete cell type assignments. For each observed gene expression profile, Pearson correlation coefficients are computed against all reference signatures in **S**. The resulting similarity scores are then normalized with the ${\rm{Softmax}}$ function to obtain probability distributions over cell types. The cell type label is defined as the index corresponding to the maximum probability. This scheme ensures that the assigned labels capture the degree of similarity between observed expression patterns and known reference signatures, thereby providing biologically meaningful supervision signals for training.

To incorporate the information from the reference signatures, we introduce a cell type classifier operating on the latent representation:

8 $${{\bf{c}}}_{n}={\rm{Softmax}}({f\!}_{{\rm{classifier}}}({{\bf{z}}}_{n};{\theta }_{c})),$$

where **z**_*n*_ is the latent representation vector, *f*_classifier_ is a neural network parameterized by *θ*_*c*_, and ${{\bf{c}}}_{n}\in {{\mathbb{R}}}^{K}$ denotes the predicted probability distribution over *K* candidate cell types. The inclusion of this classifier encourages the latent space to align with biologically relevant cell type distinctions as defined by the reference signatures.

Training of the Sig-ZIB-VAE proceeds by minimizing a composite loss function that balances three key components: (1) reconstruction fidelity, (2) latent space regularisation via the Kullback–Leibler (KL) divergence, and (3) classification accuracy with respect to correlation-derived labels. This is formalized in Eq. (9):

9 $${\mathcal{L}}={{\mathcal{L}}}_{{\rm{recon}}}+{\lambda }_{{\rm{KL}}}{{\mathcal{L}}}_{{\rm{KL}}}+{\lambda }_{{\rm{class}}}{{\mathcal{L}}}_{{\rm{class}}},$$

where the hyperparameters *λ*_KL_ and *λ*_class_ control the relative weighting of the KL and classification terms. The reconstruction component ensures that essential information in the observed expression profiles is preserved. Given the zero-inflated nature of transcriptomics data, we model the likelihood using a mixture of a point mass at zero and a Beta distribution for positive entries. The corresponding negative log-likelihood is decomposed according to Eq. (10c), where the indicator function ${\mathbb{I}}(\cdot )$ is used to separate the zero and nonzero cases and *λ*_*zero*_ is used to further control the weighting of the zero-loss term:

10a $${{\mathcal{L}}}_{{\rm{recon}}}={{\mathcal{L}}}_{{\rm{zero}}}+{{\mathcal{L}}}_{{\rm{Beta}}},$$

10b $${{\mathcal{L}}}_{{\rm{zero}}}=-{\lambda }_{{\rm{zero}}}\mathop{\sum }\limits_{n,p}\left[{\mathbb{I}}({x}_{np}=0)\log {\pi }_{np}+{\mathbb{I}}({x}_{np} > 0)\log (1-{\pi }_{np})\right],$$

10c $${{\mathcal{L}}}_{{\rm{Beta}}}=-\mathop{\sum }\limits_{n,p}{\mathbb{I}}({x}_{np} > 0)\log {p}_{{\rm{Beta}}}({x}_{np};{\alpha }_{np},{\beta }_{np}),$$

The KL divergence term - provided in Eq. (11) regularises the approximate posterior *q*_*ϕ*_(**z**_*n*_∣**x**_*n*_) to remain close to the standard Gaussian prior, thereby promoting a smooth and disentangled latent space. This encourages global structure in the latent representation, facilitating generalisation and interpretability.

11 $${{\mathcal{L}}}_{{\rm{KL}}}={D}_{{\rm{KL}}}\left({q}_{\phi }({{\bf{z}}}_{n}| {{\bf{x}}}_{n})\,\parallel \,{\mathcal{N}}(0,I)\right).$$

Finally, the classification loss component explicitly guides the latent space to align with biologically meaningful cell type distinctions derived from the signature matrix **S**. Pseudo-labels are generated by correlating each expression profile **x**_*n*_ with all reference signatures, followed by softmax normalisation and maximum selection:

12 $${y}_{n}=\arg \mathop{\max }\limits_{k}\,{\rm{softmax}}\,{\left({\rm{Corr}}({{\bf{x}}}_{n},{\bf{S}})\right)}_{k}.$$

The classification loss then penalises discrepancies between the classifier predictions and these correlation-derived labels as seen in Eq. (13):

13 $${{\mathcal{L}}}_{{\rm{class}}}=-\mathop{\sum }\limits_{c}{{\bf{1}}}_{\{{y}_{n}=c\}}\log {p}_{\psi }({y}_{n}=c| {{\bf{z}}}_{n}),$$

where *p*_*ψ*_(*y*_*n*_∣**z**_*n*_) is the classifier’s output distribution over cell types. Classification accuracy, used to evaluate model performance, is computed as the proportion of samples for which the predicted label $\arg \mathop{\max }\limits_{k}{p}_{\psi }({y}_{n}=k| {{\bf{z}}}_{n})$ matches the pseudo-label *y*_*n*_ derived from the signature correlation procedure.

By jointly optimising reconstruction fidelity, latent space regularisation, and signature-consistent classification, the composite loss provides a principled framework for analysing zero-inflated expression data. Moreover, the incorporation of correlation-derived labels ensures that the model captures interpretable, biologically grounded cell type distinctions, which are subsequently used in downstream spatial analysis.

### Spatial arrangement analysis

The work presented here further extends the OxC classification system to spatial transcriptomics by leveraging spatial neighbourhood graphs to analyse the spatial patterns and interactions between the different cell types across tissues. Here, spatial neighbour relationships were computed based on Euclidean distances, using a fixed radius approach (30*μm*). Each cell was represented as a node in a spatial graph, with edges denoting cell-cell interactions. Edges were established between nodes if their corresponding cells lay within the fixed radius. A total of 98 FOVs, measuring 0.9 mm × 0.6 mm were selected, representing a total number of 58 patients (see Supplementary Material in ref. ^9^). Following the graph computation for all available FOV, a multi-metric approach was employed toquantify the tissue architecture, using four complementary metrics.

#### Neighbourhood enrichment

Firstly, neighbourhood enrichment analysis was used to evaluate whether pair of cells are spatially adjacent more or less frequently then expected by chance. Cell-cell associations are inferred from spatial connectivity graph, where each node represents a cell and edges encode proximal spatial relationships. For each pair of cell types *i* and *j*, the observed number of neighbouring interactions *x*_*ij*_ is computed bysumming the number of edges connecting nodes of these two types. To assess statistical significance, the cell type labels are randomly permuted across the graph (typically 1000 times), while preserving the underlying connectivity structure. The mean *μ*_*ij*_ and standard deviation *σ*_*ij*_ of *x*_*ij*_ under the null distribution are estimated from these permutations. A z-score is computed for each cell type pair:

14 $${Z}_{ij}=\frac{{x}_{ij}-{\mu }_{ij}}{{\sigma }_{ij}}$$

This z-score reflects the degree of enrichment (positive values) or depletion (negative values) of interactions between cell types *i* and *j* relative to random expectation, indicating whether their spatial co-occurrence is statistically significant^21^.

#### Co-localization tests

Spatial pairwise co-localization patterns were assessed using the co-localization quotient (CLQ)^22^. *CLQ*_*a*→*b*_ is a quantitative representation of spatial co-occurrence of ‘target` and ‘infiltrating` cell types, denoted as *a* and *b*, respectively. The *CLQ* captures the degree of cell type *b* co-localizing with cell type *a* as a ratio of the observed versus the expected number of cell-type *b* within a radius of 30*μm* of cell type *a*. The *CLQ* is mathematically defined as:

15 $$CL{Q}_{a\to b}=\frac{{C}_{b\to a}/{N}_{a}}{{N}_{b}/(N-1)}$$

where *C*_*b*→*a*_ is the number of cell type *b* within a 30 μm radius of cells of cell type *a*. *N* is the total number of cells in a tissue sample, and *N*_*a*_ and *N*_*b*_ denote number of cells of cell type *a* and cell type *b* respectively. *CLQ* values were calculated for malignant cells infiltrating Fibroblast and Endothelial cells.

#### Ripley’s L statistics

Ripley’s L function is a second-order spatial statistic used to assess whether a set of spatial points exhibits clustering, regularity (dispersion), or complete spatial randomness at a given spatial scale. It provides a scale-normalized transformation of Ripley’s K function, facilitating interpretation across different radii. The L function is defined as:

16 $$L(t)=\sqrt{\frac{K(t)}{\pi }}$$

where the K function is given by:

17 $$K(t)=\frac{1}{\lambda }\mathop{\sum }\limits_{i\ne j}\frac{I({d}_{ij} < t)}{n}$$

In Eq. (17), *d*_*ij*_ denotes the Euclidean distance between points *i* and *j* in a dataset of *n* points and *t* is the spatial scale (radius) of interest. The term *λ* represent the average point density, typically estimated as *λ* = *n*/*A*, where *A* is the total area encompassing all points. The indicator function *I*( ⋅ ) equals 1 when the condition is true (i.e., when two points lie within distance *t*), and 0 otherwise. To summarize the overall spatial behavior of each cell type across all scales, we computed the area under the Ripley’s L function curve (AUC). This approach captures the cumulative extent of spatial aggregation or dispersion exhibited by a cell type across distances. Higher AUC values reflect stronger or more persistent spatial clustering over the evaluated distance range.

#### Degree centrality metrics

The final spatial metric computed in this work is represented by degree centrality^23^, which is a graph-theoretic metric, that quantifies the extent to which a cluster is connected to the rest of the network. In the context of spatial organisation, it can be used to assess how integrated or isolated a particular cluster of cells is relative to its surrounding environment.

Let *G* = (*V*, *E*), denote an undirected graph, here *V* is the set of nodes (or cells in the case of this work) and E is the set of edges representing pairwise correlations or spatial properties. Let *S* ⊆ *V* denote a subset of nodes belonging to a specific cluster *c*_*s*_. Define *N*(*S*) as the set of all nodes in the graph that are neighbours of any node in *S*, including intra-cluster and inter-cluster connections.

Degree centrality of cluster *S* is then defined as the fraction of nodes outside the cluster that are directly connected to at least one member of *S*, as described in Eq. (18).

18 $${C}_{def}(S)=\frac{| N(S)-S| }{| V| -| S| }$$

Here ∣*N*(*S*) − *S*∣ represents the number of non-cluster nodes that are adjacent or functionally integrated within the tissue, suggesting greater interaction with other clusters. Conversely, lower values suggest spatial or functional isolation, though it should be noted that this metric is inherently sensitive to cluster size and cell type abundance, as larger or more prevalent populations have greater opportunity to establish inter-cluster connections.

#### Boundary connectivity decomposition

To characterise which cell populations contribute to the inter-cluster connectivity captured by degree centrality, the cell-type composition of heterotypic neighbours was directly tallied from the spatial graph. For each FOV, all non-focal cells directly connected to at least one focal cell were identified. The cell-type composition of these boundary neighbours (observed proportion) was compared to the overall non-focal tissue composition (expected proportion) to obtain an observed-to-expected (O/E) ratio for each cell type, where values greater than one indicate over-representation at cluster boundaries and values less than one indicate under-representation. This compositional analysis complements the pairwise CLQ metric by providing a simultaneous breakdown of boundary contacts across all cell types, and is distinct from the permutation-based neighbourhood enrichment z-scores described above, which test whether the *total number* of heterotypic edges between two populations exceeds random expectation across the entire FOV.

### Statistics and reproducibility

Unless otherwise specified, all statistical tests comparing spatial metrics derived from the methods described above were performed using GraphPad Prism (version 10.5.0)^24^. For transparency, biological replicates were independent tumours. When multiple fields of view were available per tumour, spatial metrics were averaged to obtain a single patient-level estimate.

### Survival modeling

The survival analysis incorporated clinical, genomic, and spatial features to assess their prognostic significance in ovarian cancer outcomes. The baseline clinical features included the age at diagnosis, the stage of the disease and whether subjects were under treatment with neoadjuvant chemotherapy. In addition, the baseline model also included BRCA1/2 mutation status, together with the tumour mutational burden (TMB) score. Post diagnosis treatment variables including bevacizumab combination therapy and PARP inhibitor treatment were excluded from the primary baseline model to avoid treatment selection bias, as these represent post-diagnostic treatment decisions, rather than baseline prognostic attributes. For clarity, the baseline survival features were extracted from ref.^9^. Next, the baseline model was further augmented with spatiomolecular information. This included the overall proportion of EMT cells, as well as the spatial organisation metrics presented in Methods. Survival analysis was performed using the *scikit-survival* package^25^, where time-to-event outcomes were specified using overall survival as the primary endpoint, with follow-up time measured in days from diagnosis to death or last follow-up.

Spatial metrics were computed independently for each FOV as described in Methods. For patients with multiple FOVs, metric values were averaged to obtain patient-level summary statistics, yielding a single value per spatial feature per patient. A total of 20 candidate spatial features were evaluated (Supplementary Table 13), comprising: (i) Ripley’s L AUC for 7 cell populations (EMT, monocytes, T/NK cells, B cells, mast cells, fibroblasts, endothelial cells); (ii) degree centrality at 30 μm for the same 7 cell types; and (iii) pairwise metrics between EMT cells and six stromal/immune populations (neighborhood enrichment z-scores and CLQ values for EMT-monocyte, EMT-T/NK, EMT-fibroblast, EMT-endothelial, EMT-B cell, and EMT-mast cell interactions). Features with >40% missing values were excluded, and remaining missing values were imputed using median imputation.

A hierarchical modeling approach was employed to systematically evaluate the prognostic contribution of different feature sets. The analysis proceeded in four sequential stages: (1) a baseline clinical model incorporating age, disease stage, neoadjuvant chemotherapy status, BRCA1/2 mutation status, and log-transformed TMB scores; (2) an enhanced model augmenting the baseline features with EMT cell proportion; (3) spatial feature screening, in which individual spatial organisation metrics were added to the extended model to assess their independent prognostic value; and (4) construction of a final comprehensive model that integrated baseline clinical features, EMT cell proportion, and top-performing spatial features. Spatial features were retained in the final model if they met two criteria: (i) they yielded a C-index improvement over the EMT-augmented model, and (ii) they demonstrated selection stability in more than 90% of the imputed datasets, ensuring robust and reproducible associations. To prevent redundancy, pairs of highly correlated spatial features with Spearman’s correlation coefficients ≥0.85 were identified and only one feature from each pair was retained for analysis.

Moreover, to validate the prognostic significance of EMT cell proportions, patients were stratified into EMT-high and EMT-low groups using the median EMT score, consistent with the OxC framework. Kaplan–Meier survival curves with log-log confidence intervals were generated, and differences between groups were tested using the log-rank method to determine whether the survival trends previously reported in the OxC study could be reproduced in this spatially characterized dataset.

To evaluate the prognostic contribution of clinical and spatial covariates, penalized Cox proportional hazard models were implemented using elastic net regularisation. Missing data in the clinical variables were first addressed using Multiple Imputation by Chained Equations (MICE) with 20 imputed datasets. Categorical variables were numerically encoded for imputation: age (≤65 = 0, >65 = 1), disease stage (III = 3, IV = 4), neoadjuvant chemotherapy status (Untreated = 0, Treated = 1), and BRCA mutation status (Benign = 0, Pathogenic = 1). tumour mutational burden was log-transformed ($\log ({\rm{TMB}}+0.01)$) prior to imputation. The iterative imputation was performed using Bayesian Ridge regression^26^, for a maximum of 100 iterations. Finally, imputed categorical values were constrained to valid ranges and rounded to maintain categorical integrity.

The elastic net penalty combines *l*_1_ (LASSO) and *l*_2_ (Ridge) regularisation, by optimizing the penalized partial likelihood:

19 $$\arg \mathop{\max }\limits_{\beta }\,\log PL(\beta )-\alpha \left(r\underbrace{\mathop{\sum }\limits_{j=1}^{p}| {\beta }_{j}| }_{{\ell }_{1}term}+\frac{1-r}{2}\underbrace{\mathop{\sum }\limits_{j=1}^{p}{\beta }_{j}^{2}}_{{\ell }_{2} term}\right)$$

where *PL*(*β*) is the partial likelihood function of the Cox model, *β*_1_, …, *β*_*p*_ are the coefficients for *p* features and *α* ≥0 is the hyperparameter that controls the amount of shrinkage. Here, *r* ∈ [0, 1] was used to control the relative ratio between *l*_1_ and *l*_2_ penalties, hence combining the subset selection property of the LASSO penalty with the regularisation strength of the Ridge penalty. In the case of this work, the relative ratio was set to *r* = 0.05, hence imposing a more Ridge-like penalty, while retaining some variable selection capability. The optimal regularisation parameter *α* was selected through out-of-bag bootstrap cross-validation using 200 bootstrap replicates across 100 candidate *α* values spanning the range [0.01 *α*_*max*_, *α*_*max*_], where *α*_*max*_ is automatically determined by the algorithm. For each imputed dataset, predictor variables were standardized prior to model fitting, with *α* selected by maximizing out-of-bag Harrell’s C-index. Model performance and coefficients were pooled across 20 imputed datasets to obtain robust estimates that account for both model uncertainty and imputation variability.

## Results

### Sig-ZIB-VAE model performance and validation

To rigorously assess the proposed Sig-ZIB-VAE deconvolution method, data were partitioned into stratified training and validation sets, with 20% of the data held out for validation purposes. To examine model stability and reproducibility, ten independent training runs were performed using different random initializations.

**Fig. 3:**
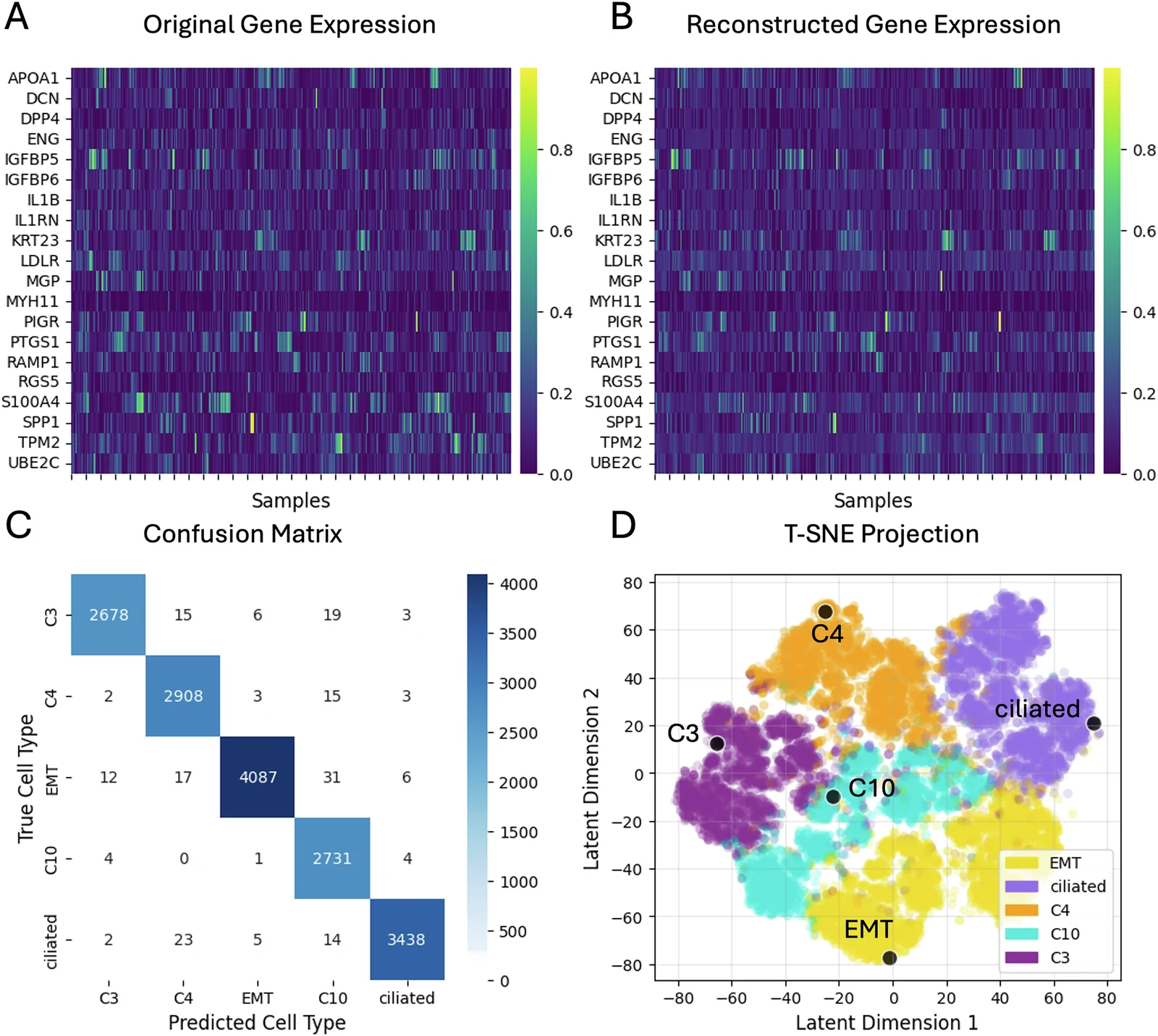
Sig-ZIB-VAE Performance. **A** Original gene expression heatmap; **B** Reconstructed gene expression demonstrating high fidelity. Expression levels are indicated by the colormaps on the right of the gene panels; **C** Confusion matrix showing the classification performance across all cell types; **D** t-SNE projection colored by predicted cell types, revealing distinct clustering. Signature cells are represented by the black dots.

The model demonstrated robust performance with mean absolute validation error of 4.03% ± 0.38% and classification accuracy of 96.54% ± 0.42% across all runs. The narrow standard deviations indicate consistent convergence independent of initialization, supporting the stability of the multi-objective optimization framework. The optimal model (selected based on lowest validation loss) achieved 3.7% reconstruction error and 96.57% classification accuracy on held-out data. The reconstruction and classification performance of the selected model can be seen in Fig. 3B, C. We also obtained comparable results to using CIBERSORTx^13^ (Supplementary Information, Section 1 and Supplementary Fig. 5).

Analysis of the learned distributional parameters revealed successful adaptation to gene-specific expression patterns (as shown in Supplementary Figs. 1, 2). Genes with high sparsity (such as DDP4, MYH11 or RGS5, for example) showed elevated zero-inflation probabilities (*π* > 0.3), while their Beta distributions, when expressed, captured the full range of possible values. Conversely, more constitutively expressed genes (such as IGFBP6, TPM2 or UBE2C) exhibited lower zero-inflation probabilities, with Beta parameters that appropriately modeled their expression distributions. Notably, beta parameters across all genes showed considerable variation, reflecting the diversity of expression patterns in the dataset.

Latent space analysis using t-SNE projection revealed distinct cell type segregation (Fig. 3D). Four of the five reference signatures were positioned at the periphery of their respective clusters, representing archetypal profiles of pure cell states. The C10 signature occupied a more central location; while this could reflect its biological role as a transient, proliferative state bridging other epithelial programs^6^, it is important to note that t-SNE is a stochastic, non-linear dimensionality reduction method whose fine-scale topology should not be over-interpreted. The central positioning may also partly arise from partial overlap among transcriptional programs in the reference signature matrix (Fig. 1B), where proliferative gene expression features are shared across C10, C3, and EMT subtypes. To formally assess the stability of the latent space and rule out stochastic visualization artifacts, we performed a complementary seed-sensitivity analysis (Supplementary Fig. 3). Briefly, t-SNE projections generated using multiple random initializations exhibited consistent global structure after Procrustes alignment^27,28^, with stable inter-cell type relationships. Moreover, a seed-independent PCA visualization reproduced the same qualitative organisation of cell types.

More robust evidence that the latent space preserves biologically meaningful structure comes from gene-specific expression gradients across the t-SNE projection (Supplementary Fig. 4). Genes exhibit spatially structured patterns that align with expected cell type-specific transcriptional programs: for example, UBE2C (a cell cycle marker) shows concentrated high expression in the C10 region, the high expression of PTGS1 overlaps the C3 cluster boundaries, PIGR localizes to the C4 cluster, TPM2 (a mesenchymal marker) concentrates in the EMT cluster, while KRT23 expression is enriched in the ciliated cell region cluster. These continuous expression gradients provide direct, gene-level validation that the learned latent representations capture and organize cells according to their transcriptional programs, independent of t-SNE’s stochastic visualization properties.

### Spatial analysis

**Fig. 4:**
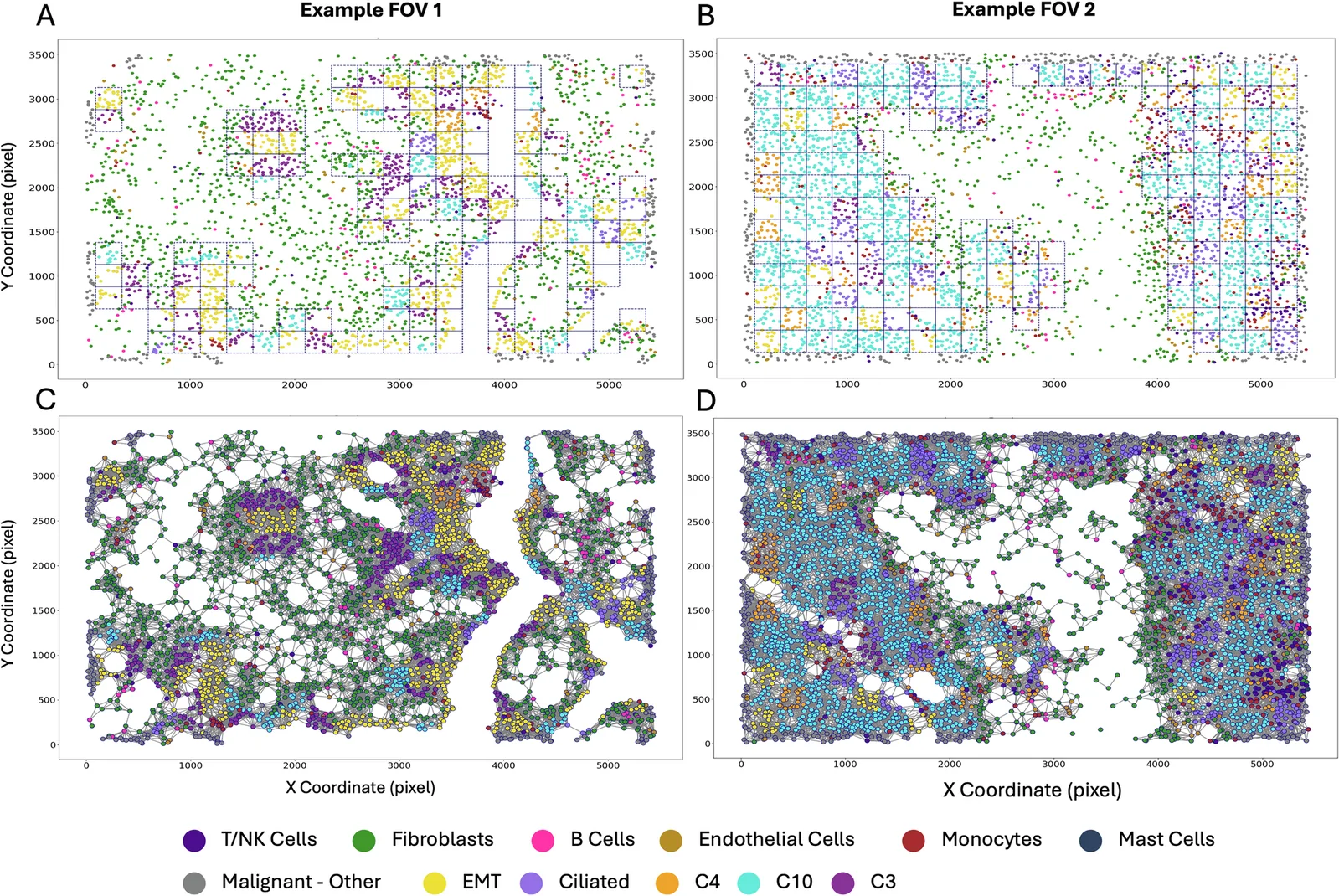
Spatial arrangement of cells across 2 fields of view (FOVs) from unique individuals. **A** and **B** show the grid used for cell aggregations across both examples; **C** and **D** show the corresponding spatial graphs. Cells are represented as nodes, while neighbourhood relations between cells are represented as edges.

**Fig. 5:**
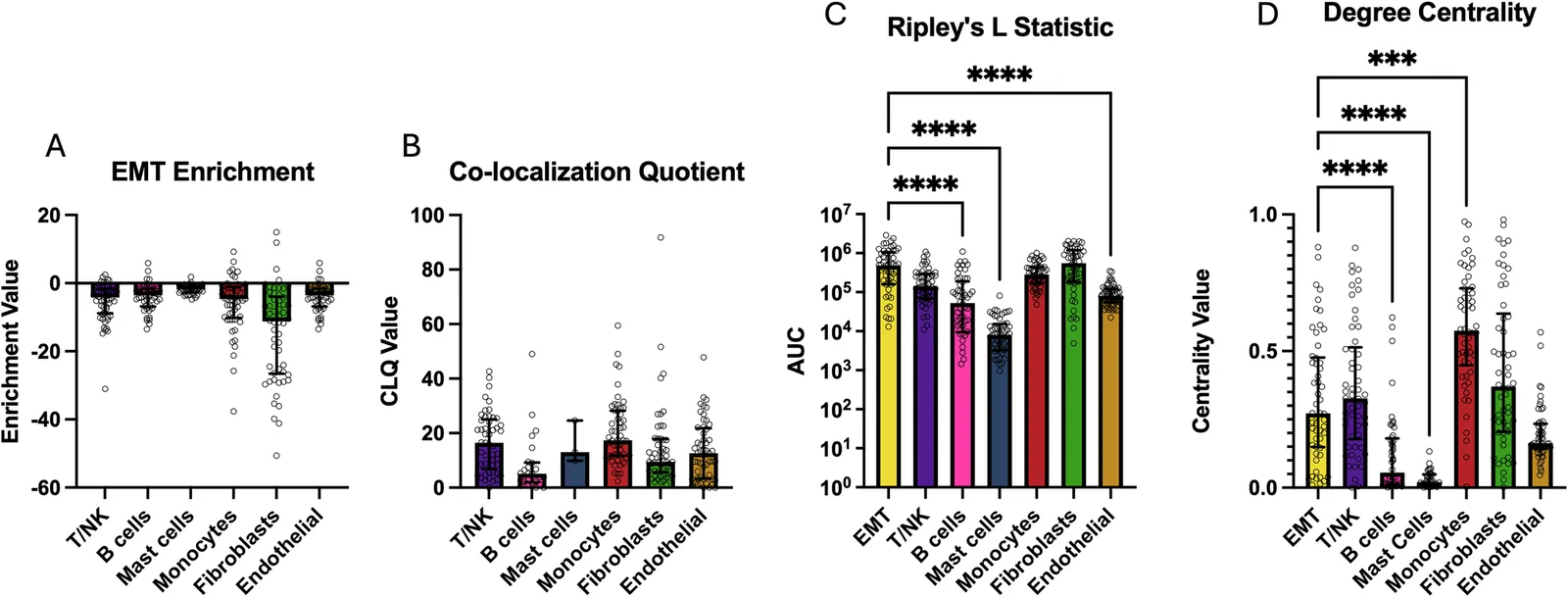
Distribution of spatial organisation metrics across the full cohort. Scatter dot plots show the overall distributions of **A** neighborhood enrichment values (n_T/NK_ = 54, n_B_ = 48, n_Mast_ = 38, n_Monocytes_ = 54, n_Fibroblasts_ = 54, n_Endothelial_ = 48 biologically independent tumours), **B** co-localization quotients (CLQ) (n_T/NK_ = 49, n_B_ = 25, n_Mast_ = 3, n_Monocytes_ = 52, n_Fibroblasts_ = 51, n_Endothelial_ = 53 biologically independent and **D** degree centrality (n_EMT_ = 54, n_T/NK_ = 54, n_B_ = 48, n_Mast_ = 38, n_Monocytes_ = 54, n_Fibroblasts_ = 54, n_Endothelial_ = 54 biologically independent tumours) across major immune and stromal cell types. Individual data points represent biologically independent tumours. Bars indicate the median; error bars denote the interquartile range (25th–75th percentiles). Statistical significance was determined using two-sided Kruskal–Wallis tests followed by Dunn’s multiple comparisons test, with a significance threshold value of *α* = 0.05. Significant differences are indicated as: *p* < 0.001 (***), *p* < 0.0001 (****).

Figure 4 shows two example fields of view (FOVs) of FTE tissue architectures, where the five OxC molecular signatures were inferred using the proposed multi-task Sig-ZIB-VAE framework. It is important to note that *Malignant - Other* cells, which are primarily located along the periphery of the FOV, were intentionally excluded from the aggregation process and were not used in any downstream analysis. This exclusion arises from the centering of spatial grids on each FOV, a deliberate design strategy aimed at reducing border effects and maintaining uniform spatial coverage across all samples^29^. Following spatial neighbourhood graph construction, the spatial organisation of EMT cells within HGSOC tissues was examined using neighbourhood enrichment, CLQ values, Ripley’s L AUC, and degree centrality metrics, as previously described. Formal definitions of these metrics are provided in the Methods section, with a concise overview presented in Fig. 5.

Firstly, neighbourhood enrichment analysis was used to quantify whether pairs of cell types are spatially adjacent more or less frequently than expected by chance, using 1000 random permutations while preserving the underlying graph connectivity and computing a z-score. Across all comparisons, EMT cells displayed negative enrichment scores, indicating consistent spatial depletion relative to random expectation (Supplementary Table 1). Collectively, these results demonstrate that predicted EMT cells occupy spatially isolated or stromal-depleted regions, showing minimal adjacency to fibroblast, endothelial, or immune compartments. The strongest depletion occurred in relation to fibroblasts (median = −11.23), followed by monocytes (median = −4.62) and T/NK cells (median = −4.22). Endothelial and B cells exhibited moderate depletion (median = −3.58 each), while mast cells were least affected (median = −1.85; all *p* < 0.0001). It is worth noting, however, that the latter score likely reflects the overall sparsity of mast cells. These results are illustrated in Fig. 5A, where the median enrichment values for all cell classes remained below zero, confirming an overall absence of localized EMT enrichment within mixed cellular niches.

Second, cell-cell co-localisation was quantified using the co-localization quotient (CLQ), which represents the ratio between the observed and expected number of neighbouring cells within a 30*μm* radius, thereby reflecting spatial preference. Across all EMT-cell type pairs, CLQ values were greater than one, indicating frequent co-occurrence beyond random expectation (Supplementary Table 2). Among all tested cell types, EMT cells exhibited the highest co-localization with monocytes (median = 17.37, *p* < 0.0001), followed by T/NK cells (median = 16.45, *p* < 0.0001). Fibroblasts (median = 9.48, *p* < 0.0001), endothelial cells (median = 12.61, *p* < 0.0001), and B cells (median = 5.17, *p* < 0.0001) also showed significant enrichment relative to random mixing, whereas mast cells did not (median = 13.01, *p* = 0.25). These findings suggest that EMT-like cells are not randomly dispersed within the tissue but display selective spatial proximity to specific stromal and immune populations—particularly monocytes and T/NK cells—within otherwise depleted microenvironmental niches (Fig. 5B).

Collectively, the CLQ and neighbourhood enrichment analyses reveal a dual pattern of EMT spatial organisation. Negative enrichment scores indicate that EMT cells preferentially cluster with other EMT cells rather than forming heterotypic neighborhoods with stromal or immune populations. However, positive CLQ values - particularly for monocytes (*median* = 17.37) and T/NK cells (*median* = 16.45) - demonstrate that specific cell types are nonetheless enriched within 30 μm of EMT cells. This pattern is consistent with EMT cells forming self-aggregating domains where the majority of neighbours are other EMT cells (explaining the neighbourhood depletion), while maintaining selective spatial proximity to monocytes and fibroblasts specifically at cluster peripheries or within mixed micro-environmental niches. Notably, both metrics operate at the same 30 μm spatial scale, indicating that the observed pattern reflects cellular composition preferences rather than scale-dependent spatial organisation.

Third, spatial clustering within each cell population was assessed using Ripley’s L function, computed across increasing spatial radii. To facilitate comparison across cell types, the area under the curve (AUC) of Ripley’s L was determined for each population, providing a global measure of spatial aggregation, where higher AUC values indicate stronger or more persistent clustering over distance. Across all compartments, predicted EMT cells and fibroblasts exhibited the highest AUC values (Supplementary Table 3). Monocytes showed intermediate clustering, whereas T/NK and B cells displayed comparatively weaker aggregation. Endothelial and mast cells exhibited the lowest AUC values, indicative of near-random or weakly clustered spatial distributions (Supplementary Fig. 5C). Statistical comparison of AUC values across cell types revealed significant differences among several populations (Supplementary Table 5). In particular, EMT cells were significantly more clustered than B cells, mast cells, and endothelial cells (*p* < 0.0001), while showing comparable clustering to fibroblasts and monocytes (*p* > 0.99). These findings might suggest that EMT cells and fibroblasts form localized clusters within the tissue microarchitecture, whereas immune and endothelial compartments display more dispersed distributions.

Finally, degree centrality was computed to assess the relative connectivity of each cellular compartment within the tissue spatial graph. This metric quantifies the proportion of inter-cluster connections maintained by a given population, thereby reflecting its level of spatial integration within the broader microenvironment. Across all cell types, monocytes and fibroblasts exhibited the highest centrality values (medians = 0.57 and 0.37, respectively), indicating strong inter-cluster connectivity and central positioning within the spatial network (Supplementary Table 4). EMT cells and T/NK cells showed moderate connectivity (medians = 0.27 and 0.33, respectively), suggesting partial integration with surrounding clusters. In contrast, B cells (median = 0.06) and mast cells (median = 0.02) displayed lower centrality scores, consistent with peripheral or spatially isolated distributions, while endothelial cells exhibited intermediate centrality (median = 0.16). Statistical comparison (Supplementary Tables 4, 6) revealed that EMT cells were significantly more connected than B cells (*p* < 0.0001) and mast cells (*p* < 0.0001), but less connected than monocytes (*p* = 0.0004). No significant differences were observed between EMT-like cells and fibroblasts, endothelial cells, or T/NK cells (*p* > 0.49 for all). These results indicate that EMT cells occupy an intermediate topological position within the tumour spatial network, being more integrated than sparsely distributed immune subsets, yet less connected than stromal populations acting as key structural hubs.

To further characterise which cell populations contribute to EMT inter-cluster connectivity, the cell-type composition of non-EMT neighbours at EMT cluster boundaries was compared to the background tissue composition. The majority of heterotypic connections involved other malignant subtypes (67.4%), while the remaining 32.6% involved stromal and immune populations. Among non-malignant cell types, monocytes were the only population over-represented at EMT boundaries relative to their tissue abundance (observed at 1.27-fold their expected frequency), whereas fibroblasts were under-represented (0.66-fold). The full results are provided in Supplementary Table 11.

To examine the interdependence between spatial metrics, pairwise correlations were computed across all features (Fig. 7B). The resulting matrix revealed moderate positive correlations between clustering (Ripley’s L AUC), degree centrality, and EMT-stromal co-localization indices, indicating that cell populations exhibiting strong self-aggregation also tend to be spatially integrated within the tissue network. In contrast, neighbourhood enrichment scores showed weaker or inverse correlations with these measures, reflecting their distinct sensitivity to large-scale depletion rather than local clustering. Overall, these analyses might highlight a multifaceted spatial phenotype of EMT cells, characterized by broad depletion from stromal and immune niches, yet persistent small-scale clustering and moderate inter-cluster connectivity, suggestive of selective integration within localized microenvironments.

#### EMT stratification analysis

**Fig. 6:**
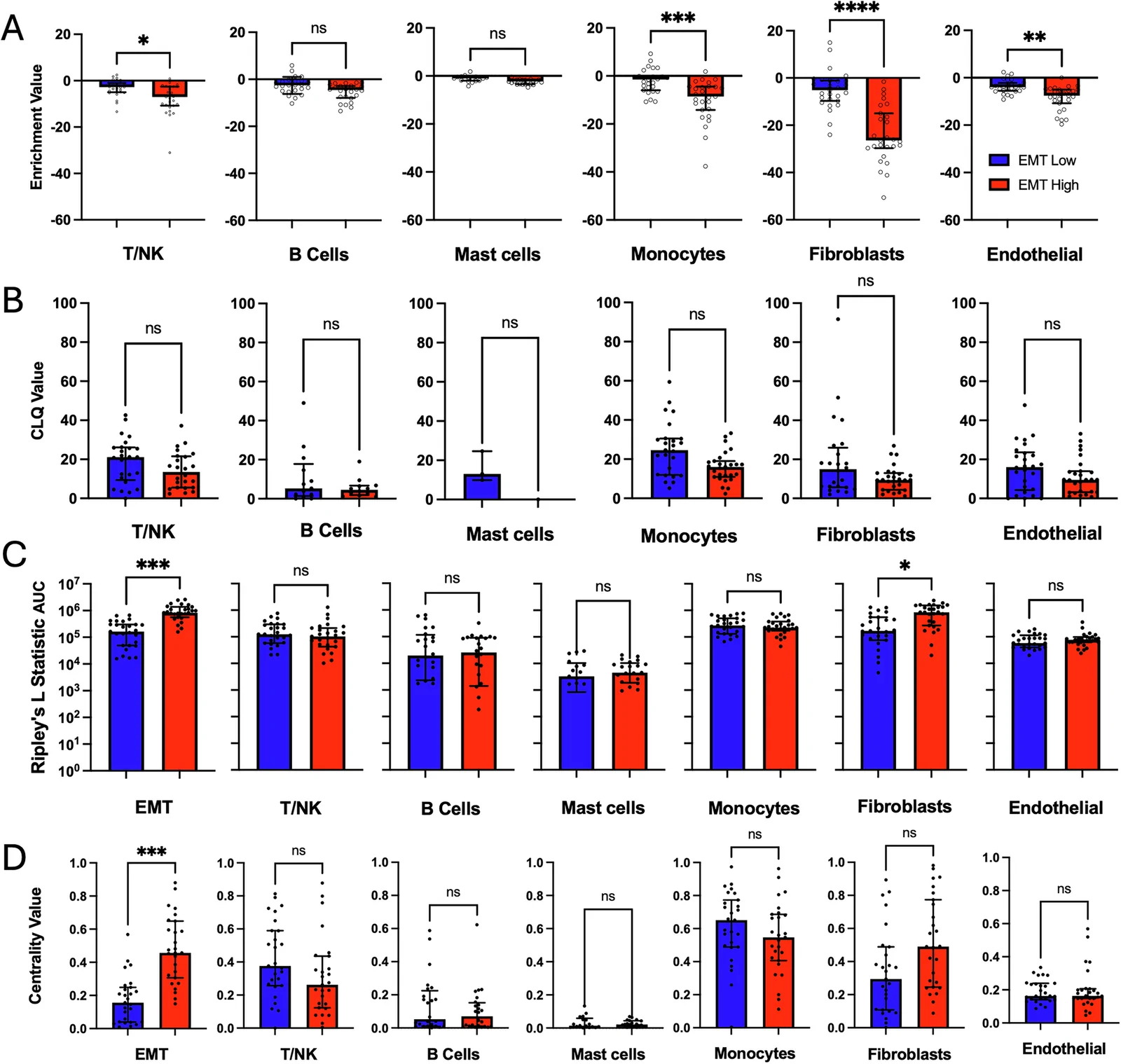
Comparison of spatial organisation metrics between EMT-high and EMT-low tumours. showing differences in neighborhood enrichment (**A**), cell–cell co-localization (CLQ) (**B**), Ripley’s *L* statistic (area under the curve) (**C**), and degree centrality (**D**) across major immune and stromal cell types. Individual data points represent biologically independent tumours. Bars indicate the median; error bars denote the interquartile range (25th–75th percentiles). Statistical comparisons were performed between EMT-high (red) and EMT-low (blue) groups using two-sided Kruskal–Wallis tests followed by Dunn’s multiple comparisons test with adjustment for multiple testing (*α* = 0.05). Significant differences are indicated as: *p* < 0.05 (*), *p* < 0.01 (**), *p* < 0.001 (***), *p* < 0.0001 (****); *ns* = not significant. The number of biologically independent tumours per group (*n*_low_/*n*_high_) were as follows: **A** T/NK (27/27), B cells (24/24), Mast cells (17/21), Monocytes (27/27), Fibroblasts (27/27), Endothelial (27/27); **B** T/NK (25/24), B cells (13/12), Mast cells (3/1), Monocytes (25/27), Fibroblasts (24/27), Endothelial (26/27); **C**, **D** EMT (27/27), T/NK (27/27), B cells (24/24), Mast cells (17/21), Monocytes (27/27), Fibroblasts (27/27), Endothelial (27/27).

Figure 6 presents the statistical analysis of the spatial distribution differences between EMT-Low and EMT-High subgroups. For clarity, stratification into EMT-Low and EMT-High subgroups was performed using the median EMT proportion value, derived from the present deconvolution method, in line with the methodology described in ref.^6^. Neighbourhood enrichment showed significantly higher depletion of EMT cells from fibroblast-rich and monocyte-rich neighbourhoods in the EMT-High group (*p* < 0.0001 and *p* < 0.001 respectively), with smaller, but significant depletion of endothelial and T/NK cells (*p* = 0.0098 and *p* = 0.0119 respectively). CLQ values did not differ significantly between EMT strata for any cells, indicating preservation of small-radius co-localisation. Not surprisingly, Ripley’s L AUC was significantly increased for the EMT-high subgroup (*p* = 0.0001), reflecting the computational dependence of this metric on EMT cell abundance (Supplementary Table 5). In addition, fibroblast populations also exhibited higher Ripley’s L AUC values in EMT-High tumours (*p* = 0.0173), consistent with enhanced local aggregation of fibroblast-rich stromal domains. Finally, EMT-High samples exhibited significantly higher degree centrality values (*p* = 0.0001), reflecting a denser network of EMT-derived connections within the tissue architecture, in accordance with the mathematical definition of this metric. Centrality changes in other cellular compartments were not statistically significant, indicating that this effect is specific to the increased representation and spatial integration of EMT-like cells rather than a global reorganisation of the spatial network. Overall, these results suggest that EMT-high tumours are characterised by large-scale depletion of EMT cells from stromal and immune neighbourhoods, preservation of short-range co-localisaion, increased intra-population clustering of EMT and fibroblast cells, and higher network connectivity. These findings may indicate that EMT-high tumours undergo spatial reorganisation, marked by formation of locally clustered, mesenchymal-rich domains rather than homogeneous cellular mixing (full results tables are given in Supplementary Tables 7–10).

### Survival analysis

**Fig. 7:**
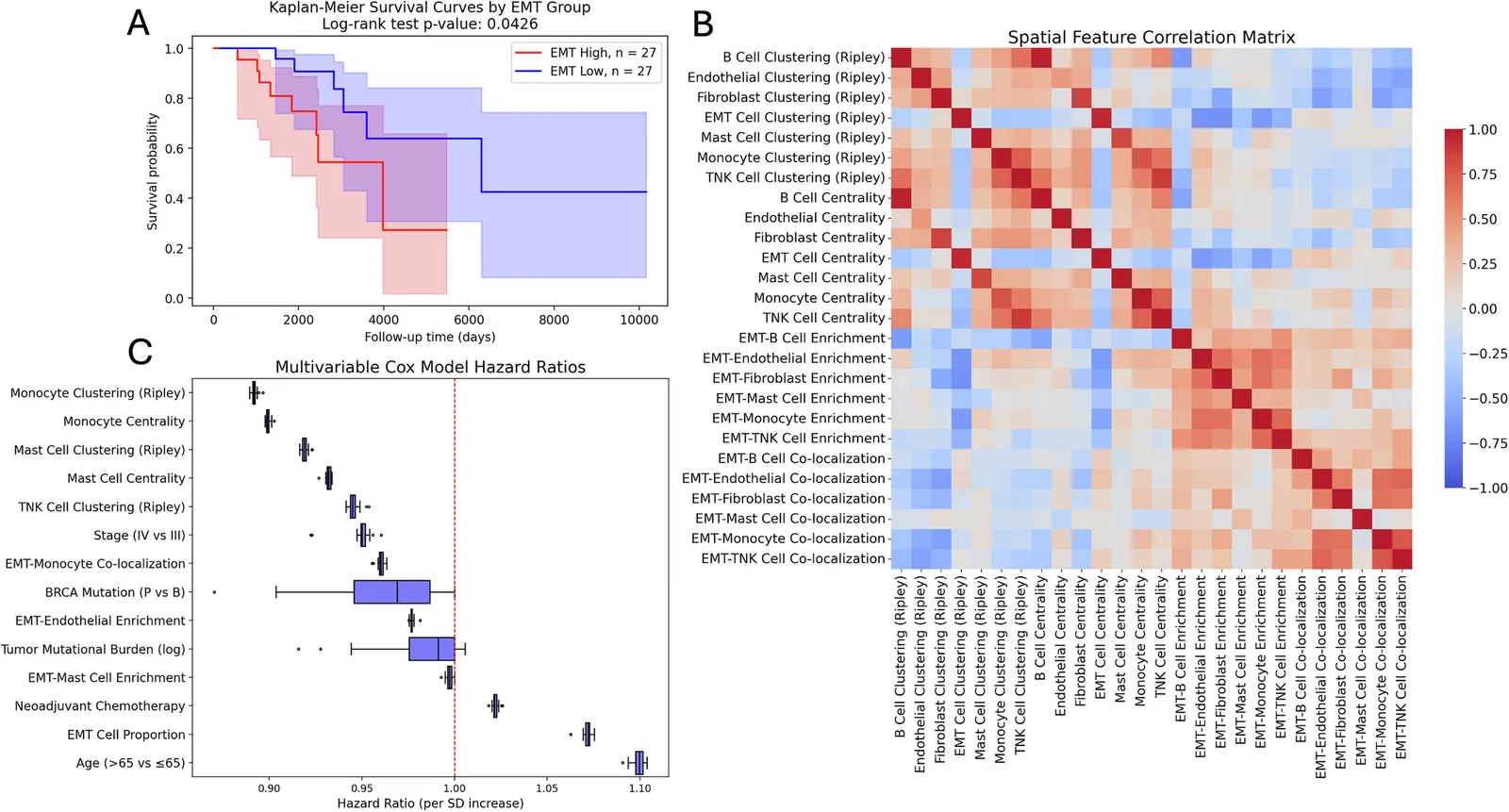
Survival analysis results. **A** Kaplan–Meier curves stratified by EMT proportion (median split; High *n* = 27 vs Low *n* = 27; log-rank *p* = 0.0426). The shaded areas denote the 95% confidence intervals. Notably, this analysis confirms that the modified EMT signature used in this study preserved the prognostic stratification originally reported in the OxC study^6^, with EMT-high tumours exhibiting significantly poorer overall survival. **B** Spearman rank correlation matrix candidate spatial tumour microenvironment features. **C** Hazard ratios (HRs) from the final penalized Cox model integrating baseline clinical variables, EMT cell proportions, and top-performing spatial features (*n* = 54 biologically independent tumours; boxplots show the distribution of HRs across *m* = 20 multiply-imputed datasets). For boxplots, boxes represent the interquartile range (25th–75th percentiles) with the median indicated by the central line; whiskers denote the 2.5–97.5 percentile range, and outliers are shown as individual points. The vertical red dashed line at unity HR denotes no association with hazard; values to the left indicate decreased risk, whereas values to the right denote increased risk.

This section presents the results of the multi-faceted survival analysis. First, Kaplan–Meier survival analysis (Fig. 7A) revealed that patients in the EMT-high group had significantly worse overall survival outcomes than those in the EMT-low group (log-rank test: *χ*^2^ = 4.11, *p* = 0.043). These results confirm that EMT-high status is associated with poor prognosis, consistent with findings from the OxC study^6^, further validating the use of the EMT proportion as an independent prognostic factor in HGSOC. Additionally, to verify that the modified 20-gene signature matrix used in this study retains the same prognostic discriminative ability as the original 52-gene OxC panel, an external validation was performed on *non-spatial* bulk-expression cohorts drawn from the original OxC study^6^, combining eight independent datasets, including The Cancer Genome Atlas (TCGA) dataset^11^, the Australian Ovarian Cancer Study (AOCS) dataset^30^ and six additional microarray datasets from the CuratedOvarianData database^31^. EMT proportions were re-estimated using the 20-gene signature matrix and dichotomised by the within-cohort median. In the pooled meta-cohort (*n* = 1657), EMT-high cases again exhibited significantly inferior overall survival (unstratified log-rank: *χ*^2^ = 20.57, *p* = 5.76 × 10^−6^; stratified by study: *χ*^2^ = 21.15, *p* = 4.24 × 10^−6^). Full details and the corresponding Kaplan–Meier curves are provided in Supplementary Section 2 and Supplementary Fig. 7.

#### Spatial metrics prognostics

As described in the Methods Section, the baseline elastic net Cox model—incorporating age, stage, neoadjuvant chemotherapy status, BRCA1/2 mutation status, and log-transformed TMB - was first tuned for regularisation strength. At the optimal penalty (*α* = 0.61), the mean out-of-bag C-index was 0.506, indicating near-random discrimination and confirming that clinical and genomic covariates alone provide limited prognostic separation in this cohort. Refitting at this penalty across all 20 imputed datasets yielded an apparent Harrell’s C-index of 0.670 ± 0.017. Incorporating EMT cell proportions raised this to 0.713 ± 0.015—an absolute gain of ~6.4%—demonstrating that EMT burden contributes independent prognostic information beyond conventional factors, with higher EMT proportions consistently associated with poorer survival (pooled HR = 1.101 ± 0.003).

Following correlation-based filtering (Fig. 7B), individual spatial metrics were systematically added to the EMT-augmented model. Monocyte spatial organisation emerged as the dominant prognostic signal: both degree centrality and Ripley’s *L*-based clustering achieved the highest discriminative performance (C-index: 0.771 ± 0.013 and 0.761 ± 0.012, respectively), with complete selection stability across imputations and hazard ratios indicating a protective association (HR ≈ 0.84–0.85). Mast cell and T/NK cell organisation provided additional, consistent prognostic information at slightly lower discrimination (C-index range: 0.731–0.743), while interaction metrics between EMT cells and immune or endothelial populations offered only marginal improvements—providing suggestive but limited evidence for the prognostic relevance of direct tumour–immune spatial relationships. Individual feature contributions are detailed in Supplementary Table 13.

In the final integrated model, spatial metrics dominated the prognostic landscape. The strongest protective effects came from monocyte organisation, with clustering and degree centrality each corresponding to an ~10–11% hazard reduction per standard deviation increase (HR = 0.89 and 0.90). Mast cell and T/NK cell spatial features contributed additional, smaller protective effects (HR = 0.92–0.95). Among non-spatial covariates, age and EMT proportion remained adverse prognostic factors (HR = 1.10 and 1.07), while stage and BRCA mutation status showed paradoxical protective associations (HR = 0.95 and 0.96), likely reflecting treatment-related confounding.

## Discussion

This paper proposed a sequential approach for extending the OxC classification framework into the spatial domain by developing a deep learning-based deconvolution method specifically tailored for spatial transcriptomics data. As such, here, we firstly introduced the Sig-ZIB-VAE model as a deep generative modeling approach for spatially aggregated gene expression data, by combining a zero-inflated Beta likelihood with amortised variational inference, thereby explicitly capturing zero-inflation, while respecting the [0, 1] support for proportion-like inputs.

It should be noted that in contrast to popular single-cell VAE-based methods that assume count distributions (such as NB/ZINB in scVI/scVAE)^18,32^, our Beta parametrisation is tailored to normalised measurements and should not be confused with the disentanglement-oriented *β*-VAE family^33^. By coupling the decoder with signature-guided supervision in the latent space, Sig-ZIB-VAE leverages prior biological knowledge, analogously to reference-driven deconvolution and annotation frameworks - such as CIBERSORT/MuSiC for bulk data and SPOTlight, Stereoscope, and cell2location for spatial assays - yet retains the benefits of a fully generative model (uncertainty-aware reconstructions, data simulation, and joint optimization of reconstruction and classification)^15,34–37^.

Compared with regression-based deconvolution approaches, the multi-task objective presented here encourages biologically structured latent space (akin to weakly supervised approaches like CellAssign/scANVI), while scaling efficiently to large datasets via mini-batch stochastic optimisation^18,38,39^. Moreover, the generative modeling approach presented here offers significant advantages for future single-cell applications. By explicitly learning the underlying gene expression data, the model can also generate pseudo-realistic samples that preserve both sparsity patterns and continuous expression characteristics. This capability enables principled data augmentation to address class imbalance issues, which are common in clinical datasets and improve model robustness to out-of-distribution samples from different experimental conditions or technologies, another critical limitation identified by Yang et al.^40^. Furthermore, the generative framework facilitates privacy-preserving data sharing and provides a pathway to develop more robust and generalizable predictive models, as single-cell studies increasingly move towards clinical applications with inherently limited samples sizes and high variability. Finally, grounding supervision in FTE-derived reference signatures aligns the representation with cell states known to stratify HGSOC, including EMT programs linked to adverse outcomes, thereby supporting downstream spatial analyses and prognostic interpretation^7^.

Following deconvolution analysis, spatial quantification of EMT cell distributions uncovered insights into tumour microenvironment organisation, revealing a non-uniform distribution of EMT cells in HGSOC tissues. The results show that EMT cells are broadly depleted from stromal and immune neighbourhoods, yet maintain persistent clustering and selective integration within the tissue network - evidenced by higher EMT and fibroblast clustering, co-localisation with selected cell populations (notably monocytes), and increased degree centrality. These findings suggest that EMT-high tumours exhibit distinct spatial organisation characterized by locally clustered, mesenchymal-rich domains rather than homogeneous cellular distribution.

The spatial organisation of the cells surveyed in this study appears to be consistent with recent spatial transcriptomic studies demonstrating that tissue architecture, beyond bulk compositional analysis, correlates with clinical outcomes, where tumour-stromal niches and cellular neighbourhoods may act as functional units of disease progression^41^. Notably, Withnell et al.citeWithnell2024 identified EMT hotspots in breast cancer that colocalize with hypoxic/angiogenic regions characterized by immune cell insulation. Although our study did not directly evaluate hypoxia-associated markers, the observed spatial distribution of EMT cells - forming dense homotypic clusters that are broadly depleted from stromal and immune neighbourhoods yet maintain selective monocyte co-localization at their peripheries - suggests that similar microenvironmental pressures may underlie spatially compartmentalized mesenchymal programs in HGSOC. However, direct validation of this hypothesis will require future study. Specifically in HGSOC, Zhang et al.^42^ revealed that changes in immune cell localization and stromal architecture are associated with early relapse, emphasizing the clinical relevance of spatial organisation beyond cellular abundance.

Beyond describing network topology, the observed spatial patterns suggest distinct biological modes of EMT organisation in HGSOC. EMT-like malignant cells were broadly depleted from stromal and immune neighbourhoods at the tissue scale, yet consistently formed locally clustered, mesenchymal-rich domains that retained selective short-range proximity to monocytes. This multi-scale phenotype argues against indiscriminate mixing and instead supports a model in which EMT programs are stabilised within spatially segregated niches. The observed clustering is consistent with prior evidence that EMT programs can retain intercellular adhesion and promote collective tumour cell behaviour rather than isolated dissemination^43,44^, a mode of organisation relevant to ovarian cancer progression where multicellular spheroids contribute to peritoneal spread^45^.

The preservation of EMT-monocyte co-localisation at short spatial radii, despite global depletion, suggests selective interactions at niche boundaries rather than within fully immune-infiltrated regions. One possible mechanism, supported by prior studies, is paracrine crosstalk between mesenchymal tumour cells and macrophage-lineage cells, in which EMT-associated programs recruit monocytes while macrophage-derived factors reinforce mesenchymal transcriptional states^46,47^. In HGSOC, tumour-associated macrophages also contribute to extracellular matrix remodelling through secretion of proteins such as TGFBI and tenascin C^48^. Conversely, the large-scale depletion of EMT cells from T/NK and B cell neighbourhoods aligns with immune exclusion phenotypes linked to EMT, including TGF-*β*-mediated restriction of cytotoxic lymphocyte infiltration^49^, consistent with the original OxC association between EMT and immunosuppression^6^.

Notably, monocyte spatial organisation - rather than EMT-monocyte proximity alone - emerged as the strongest protective prognostic feature, indicating that immune architecture plays a dominant role in shaping clinical outcomes. Higher monocyte clustering and network integration may reflect more organised immune configurations, which in other studies have been associated with improved prognosis in HGSOC^50^, whereas dispersed monocyte distributions may indicate diffuse immunosuppressive infiltration. Together, these findings reinforce the concept that spatial organisation of tumour-immune interactions, rather than cellular composition alone, is a critical determinant of disease trajectory.

Critically, the survival analysis showed that EMT-high status (i.e., greater EMT proportion) was associated with poorer prognosis—consistent with previous OxC studies^7,8^ - thereby validating EMT proportion as an independent prognostic factor in HGSOC. Moreover, the spatial organisation of immune cell populations - particularly monocytes - also emerged as a dominant determinant of outcome. Among all features, monocyte integration within their local neighborhood (captured by degree centrality) and their spatial clustering (quantified using Ripley’s L AUC) showed the strongest and most consistent prognostic associations, aside from EMT cell proportion and age covariates. Additional protective effects were observed for mast cell spatial metrics and T/NK cell clustering. This hierarchy of prognostic significance may further suggest that the spatial architecture of the tumour-immune interface supersedes cellular composition alone in determining clinical outcomes. Overall, these findings support our initial hypothesis that the OxC classification—originally derived from bulk transcriptomic data—encodes spatially meaningful biological organisation when projected onto high-resolution tissue data, thereby bridging molecular classification with spatial tissue ecology.

Having presented the outcomes of the OxC extension to the spatial domain, it is also important to acknowledge the limitations of the present work and outline potential future directions. While this analysis employed a subset of the OxC gene panel overlapping with the CosMx signatures, a natural next step would be to expand the evaluation to the full OxC repertoire to capture the complete transcriptional heterogeneity originally defined by Hu et al.^6^.

Moreover, several aspects of model evaluation warrant careful consideration. Model performance assessment employed held-out samples from the same spatial transcriptomics dataset, which, while suitable for internal benchmarking, may not fully represent the model’s generalization across independent cohorts or experimental conditions. Although the resulting high classification accuracy is encouraging, it remains derived from pseudo-labels defined by correlations with established signatures rather than ground-truth cell annotations. This circular validation approach - common to deconvolution studies - constrains the ability to quantify absolute classification performance.

Additionally, three methodological considerations affect the interpretation of spatial patterns. First, the grid-based spatial aggregation strategy used fordeconvolution may contribute to the observed degree of self-clustering, as cells within the same grid square receive identical program assignments by design. While this approach enhances statistical power for signature detection, it may amplify local homotypic clustering patterns relative to true single-cell-resolution spatial organisation, and the extent to which grid-level assignments versus genuine biological clustering drive the observed EMT self-aggregation patterns warrants further investigation using cell-level deconvolution methods. Second, given the limited scale of the analysed fields of view, the findings are best interpreted as capturing local cellular interactions rather than full tissue architecture. At this mesoscopic scale, spatial statistics reflect intra-regional patterns of cell organisation—such as clustering, adjacency, and network connectivity—but cannot resolve macro-architectural gradients across entire tumour sections. Third, while degree centrality quantifies the overall inter-cluster connectivity of a cell population, it does not distinguish which cell types constitute those connections. To address this, we tallied the cell-type composition of non-EMT neighbours at EMT cluster boundaries and compared it to the background tissue composition, revealing selective monocyte over-representation (Supplementary Table 11). The majority of heterotypic connections nonetheless involved other malignant subtypes, and alternative graph-theoretic metrics such as betweenness centrality or modularity-based community detection may provide complementary insights into the relative contributions of malignant cell mixing and immune-stromal boundary interactions^51^. Thus, identifying biologically relevant tissue regions and quantifying intercellular relationships at the appropriate spatial scale remain important open challenges for future spatially resolved studies^52^.

Furthermore, independent validation of spatial clustering and prognostic findings in external cohorts represents an important priority which is, however, currently constrained by data availability. This study represents the first attempt to extend the OxC classification framework to spatial transcriptomics, and to our knowledge, no independent spatial transcriptomics datasets currently exist that simultaneously provide: (1) HGSOC samples profiled at single-cell resolution, (2) gene panels overlapping with the OxC signature repertoire, (3) matched clinical outcomes data, and (4) comparable tissue sampling strategies (e.g., TMA-based FOVs capturing tumour microenvironmental heterogeneity). While the EMT prognostic signal was successfully validated across eight independent bulk transcriptomics cohorts (Supplementary Section 2), confirming that the modified 20-gene signature retains discriminative power, the spatial organisation patterns and spatial metric prognostics require replication in future spatial profiling studies as such datasets become available.

## Supplementary information

Supplementary Information

Description of Additional Supplementary files

Supplementary Data 1

## Data Availability

Spatial transcriptomics data were obtained from the Discovery dataset described in Yeh et al.^9^ and available on Zenodo^14^ (https://doi.org/10.1038/s41590-024-01943-5). The procedure for acquiring the signature matrix used in ref. [6] can be found on the Github repository (https://github.com/zhiyhu/scFT-paper). Source data for Supplementary Figs. 3–7 are provided in Supplementary Data 1.

## Code availability

The code used to generate the results presented in this paper is made available at a Github repository (https://github.com/cwcyau/oxc_spatial) and are also archived on Figshare^53^ (https://doi.org/10.6084/m9.figshare.31988754).
