# Supplementary material for: Understanding the spatial determinants of the Oxford Classic prognostic signature for high-grade serous ovarian cancer
Source: Commun Med (Lond). 2026 Jun 6;6:484. doi: 10.1038/s43856-026-01708-1 (PMC13574789; doi:10.1038/s43856-026-01708-1)
Supplement: Supplementary file 1 [file 43856_2026_1708_MOESM1_ESM.pdf]

Understanding the spatial determinants of the Oxford Classic prognostic signature for high-grade serous ovarian cancer - Supplementary Information

Alexandru Stihi[1] and Christopher Yau[1,2,*]

[1]Nuffield Department for Women's and Reproductive Health, University of Oxford, Oxford, UK.
[2]Health Data Research UK, London, UK.
[*]Corresponding author: christopher.yau@wrh.ox.ac.uk

May 22, 2026

Supplementary Information

1 Sig-ZIB-VAE validation checks

The comparison between the empirical distribution of the normalized gene expression data and the learned distributions using the proposed Sig-ZIB-VAE model is shown in Supplementary Figure 1. Subsequently, Supplementary Figure 2 provides further insights into the reconstruction quality. Moreover, Supplementary Figure 4 showcases the two-dimensional projection of the embedding space, highlighting the normalised expression values for individual genes. This visualization enables verification that the spatial organization of individual genes within the latent space corresponds to known cell type–specific expression patterns derived from the gene signature matrix. For example, genes such as UBE2C, which are expected to be selectively expressed in the C10 cluster based on the signature matrix, should exhibit higher expression intensity (brighter coloration) confined to the C10 region of the latent embedding, with minimal expression elsewhere. Such concordance provides qualitative evidence that the latent space representation accurately captures and preserves biologically relevant transcriptional structure across cell populations.

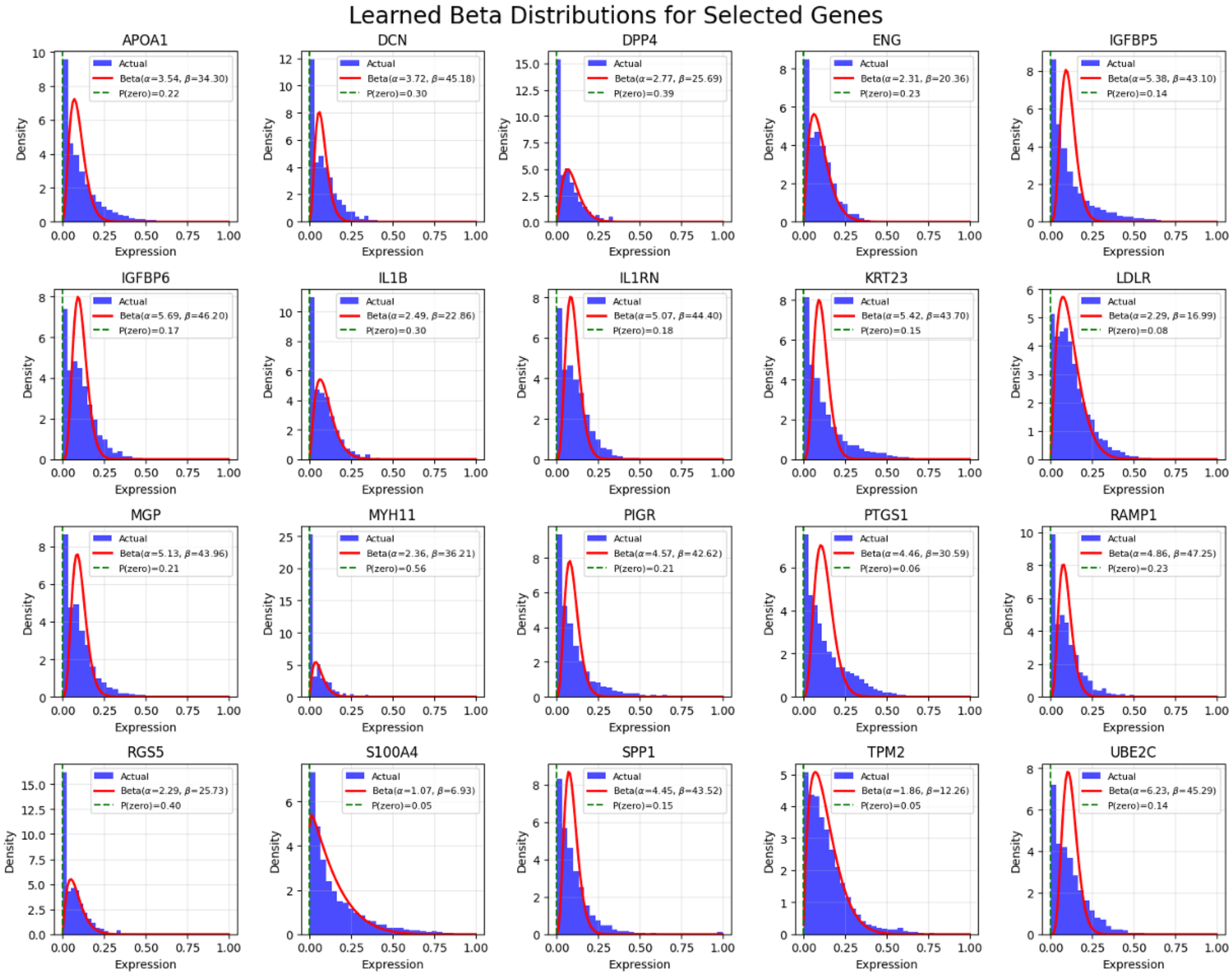

Supplementary Figure 1: Learned zero-inflated Beta distributions for normalized gene expression. For each gene, the empirical distribution of the normalised values (blue) is overlaid with the model-implied Beta density from the decoder head (red line; parameters α, β displayed in each panel). The dashed green line annotates the estimated zero-inflation probability π. The close agreement between empirical histograms and fitted densities indicates that the Zero-Inflated Beta VAE captures both the pronounced right-skew and the excess zeros characteristic of these expression profiles.

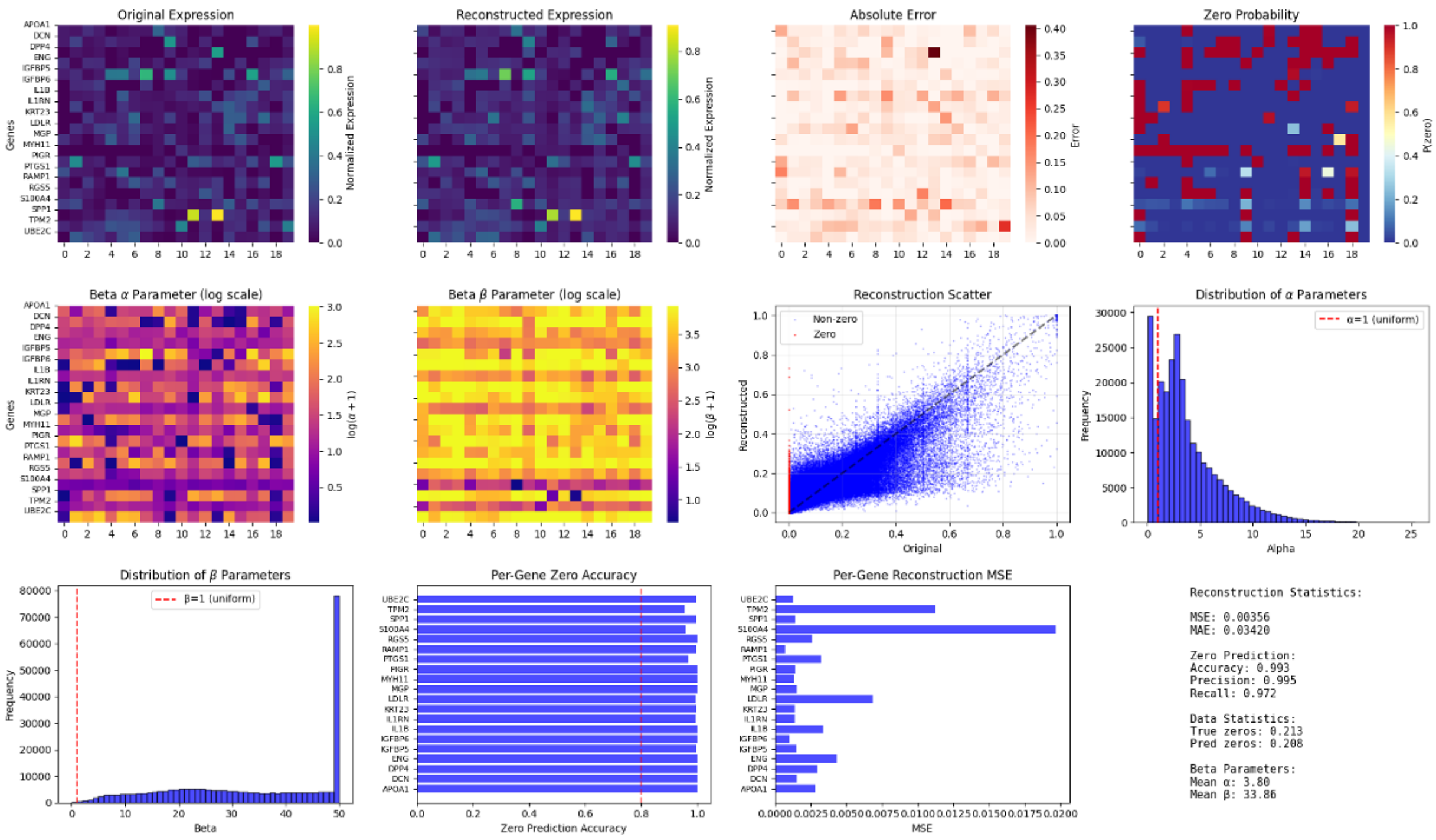

Supplementary Figure 2: Zero-Inflated Beta VAE reconstruction and parameter diagnostics. **Top row** (left to right): original normalized expression across all 20 genes, reconstructed expression, absolute error, and predicted zero probability (π). **Middle row**: decoder Beta parameters per gene/sample on log scale ($\log \alpha + 1$, $\log \beta + 1$), reconstructed vs. original scatter with the 45ř line , global α distribution. **Bottom row**: global β distribution, per-gene zero-prediction accuracy and per-gene reconstruction mean squared error (MSE).

To further evaluate the stability of the learned latent representation, the sensitivity of the t-SNE projection to random initialization was evaluated (Supplementary Figure 3). Latent embeddings were projected using seven different random seeds. To account for arbitrary rotations and reflections inherent to t-SNE, embeddings were aligned using Procrustes analysis. While absolute cell coordinates exhibited variability consistent with the stochastic nature of t-SNE (mean Euclidean standard deviation: 21.86), relative spatial relationships were highly preserved. In particular, pairwise inter–cell type distances showed a low coefficient of variation ($CV \approx 2.45$), indicating that the global structure of the latent space remains stable across initializations. The C10 signature displayed comparatively lower positional variability (Euclidean standard deviation: 7.78), supporting the robustness of its central placement.

To provide a seed-independent assessment of latent structure, we additionally performed Principal Component Analysis (PCA) on the latent representations (Supplementary Figure 3 - bottom right). The first two principal components explained 37.2% of the total variance (PC1: 21.2%, PC2: 16.1%) and reproduced the same qualitative relationships among cell types observed in the t-SNE projections, including the central positioning of C10. This concordance confirms that the observed organization reflects intrinsic structure in the learned representation rather than visualization artifacts.

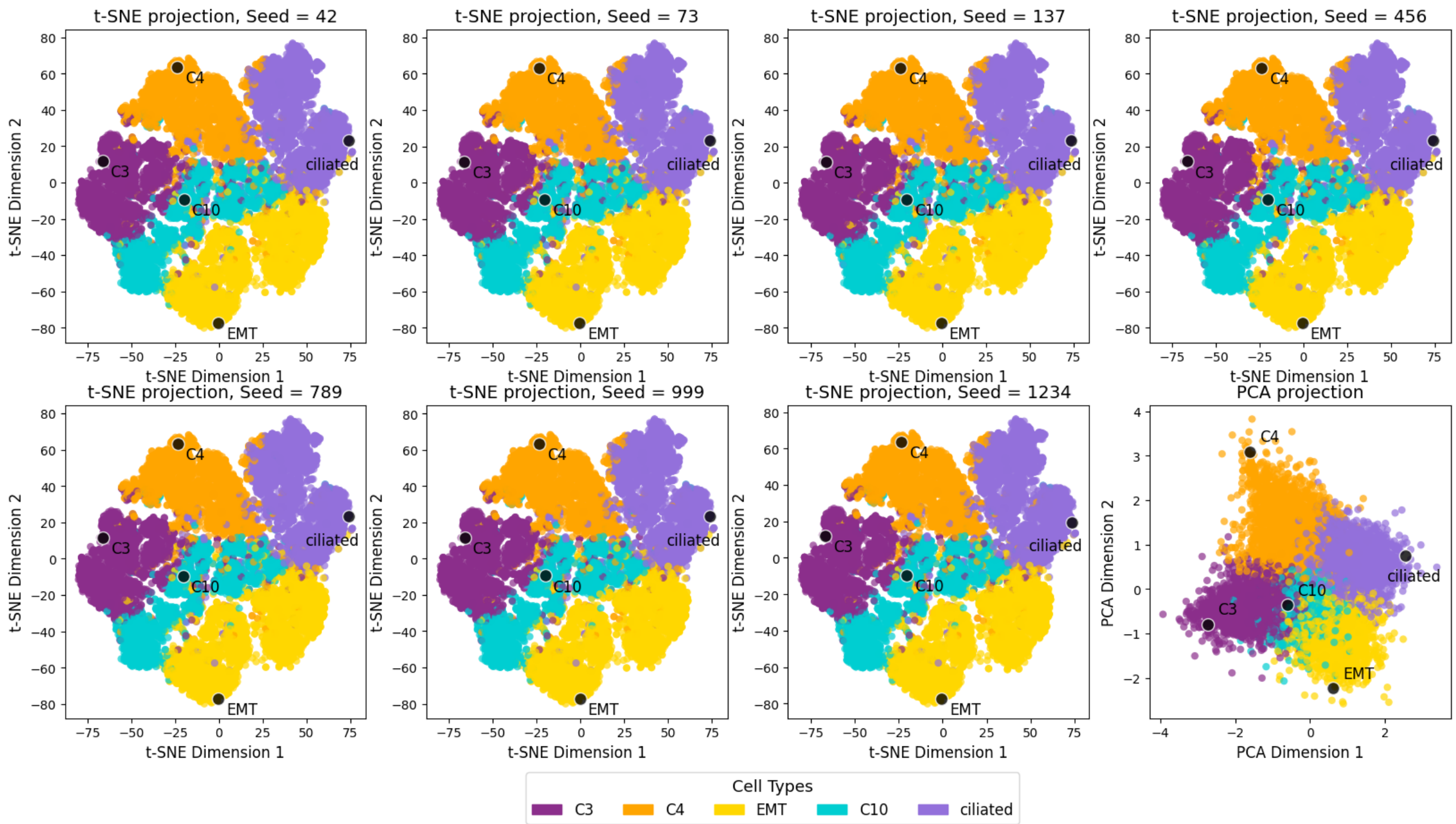

Supplementary Figure 3: Latent space stability across random initializations. t-SNE projections of the learned latent space generated using seven different random seeds (42, 73, 137, 456, 789, 999, 1234) demonstrate consistent global organization of cell types despite stochastic variation in absolute coordinates. Black dots indicate reference signature centroids. Across seeds, relative spatial relationships among C3, C4, EMT, C10, and ciliated cell populations are preserved, with C10 consistently occupying a central position. The PCA projection (bottom right) provides a seed-independent visualization and reproduces the same qualitative structure, confirming that the latent organization reflects intrinsic biological structure rather than t-SNE initialization effects.

To assess deconvolution results concordance with an established reference, per-patient program proportions estimated through the Sig-ZIB-VAE framework were compared with those obtained using CIBERSORTx [1]. The five molecular program proportions can be visualised in Supplementary Figure 5. The latter deconvolution utilised the same signature matrix and *pseudo-bulk* data generated by aggregating all cells from the same patient and calculating the mean expression per gene. Quantitative comparison of the deconvolution results yielded a mean absolute error of 13.53% and an RMSE of 18.73%. While these values reflect inherent methodological differences, they are consistent with acceptable performance thresholds for cell-type proportion estimation, suggesting that both approaches capture broadly similar biological signals. The code for deconvolution analysis using CIBERSORTx is available at `https://github.com/zhiyhu/scFT-paper`.

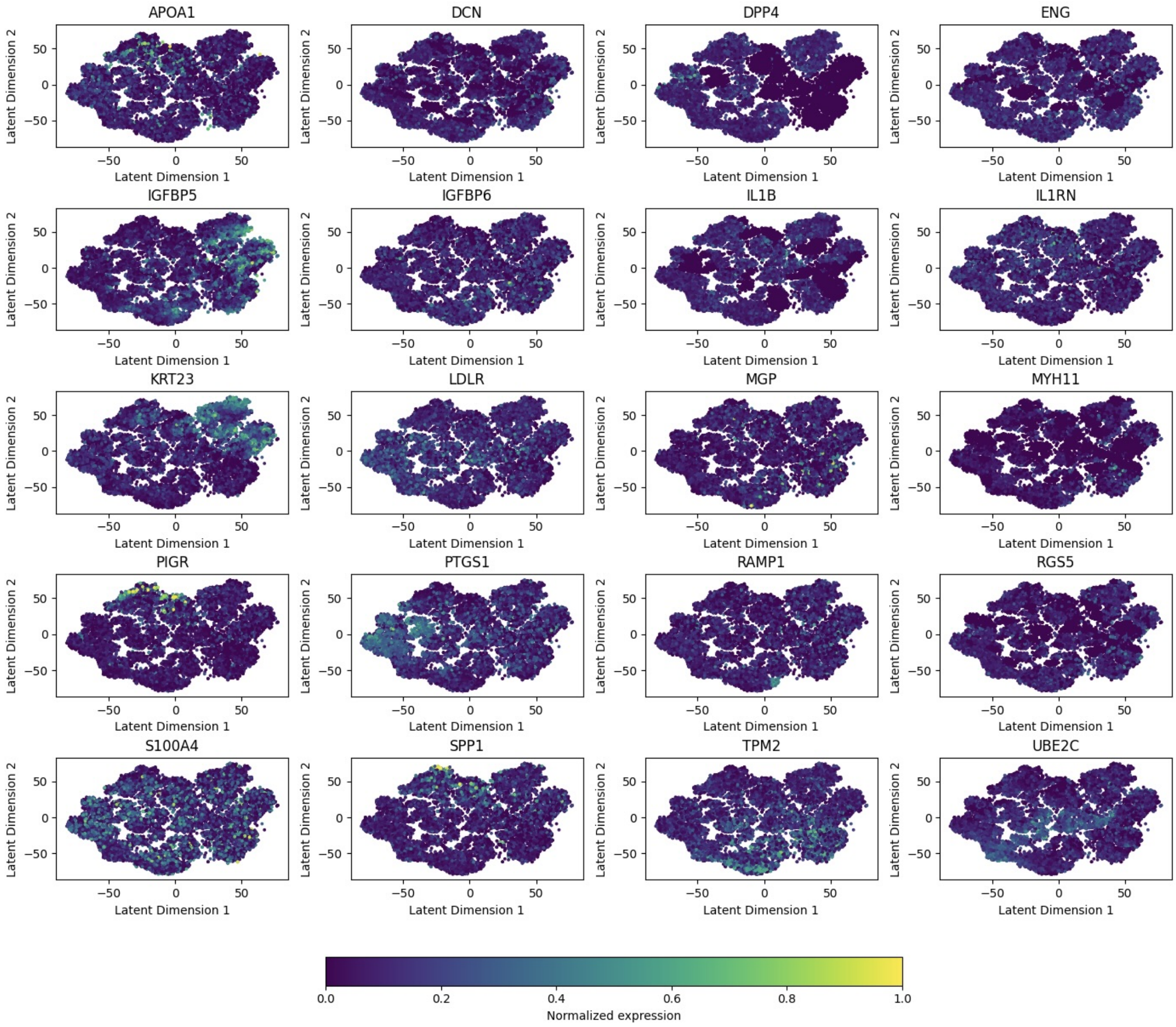

Supplementary Figure 4: Gene-specific expression patterns mapped onto t-SNE latent space projection. Each panel shows the two-dimensional t-SNE embedding of the leaned latent representations, colored by normalized expression values for individual genes. The color scale indicates expression levels from zero (dark purple) to maximum expression (yellow).

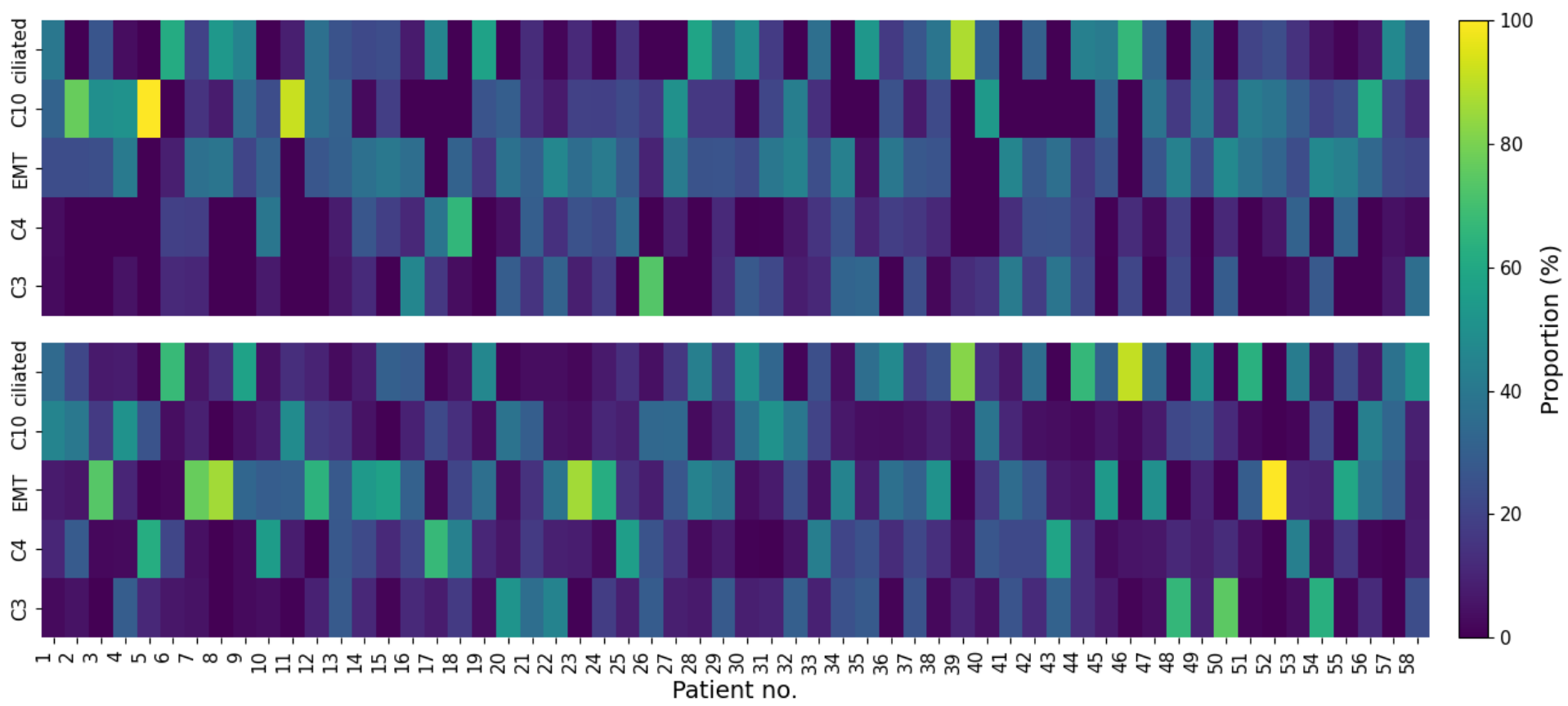

Supplementary Figure 5: Comparison of deconvolution estimates between CIBERSORT and Sig-ZIB-VAE. Heatmaps show programme proportions (0–100%) per patient (columns) for the five signatures (rows: C3, C4, EMT, C10 and ciliated). The top panel displays CIBERSORTx estimates; the bottom panel displays Sig-ZIB-VAE estimates for the same samples.

2 Signature matrix validation

To evaluate the validity of the modified 20-gene signature matrix used in this study, principal component analysis (PCA) was conducted on the TCGA dataset using the original 52-gene OxC signature and the reduced 20-gene panel independently. The results indicate that the principal biological signals captured by the full 52-gene panel are largely preserved within the reduced gene set. Specifically, the leading axes of variation exhibited strong concordance across panels: the second principal component derived from the 52-gene signature was highly correlated with the first principal component of the 20-gene panel (Pearson $r = 0.87$, $p << 0.0001$), while additional major components demonstrated moderate-to-strong correlations ($PC3_{52}$ with $PC2_{20}$: $r = 0.59$; $PC5_{52}$ with $PC4_{20}$: $r = 0.68$). Scatter plots of these matched component pairs reveal clear linear relationships (Supplementary Figure 6B–C), supporting the conclusion that the reduced panel recapitulates the dominant biological gradients identified by the full signature. In quantitative terms, the first three principal components of the 52-gene panel accounted for 50.0% of the total variance, whereas the first three components of the 20-gene panel explained 52.3% (Supplementary Figure 6D), indicating substantial retention of variance despite dimensionality reduction.

One exception is PC1 from the 52-gene panel (22.7% of variance), which captures the ciliated-versus-EMT axis - with positive loadings on canonical ciliated markers (*TEKT1, EFCAB1, SNTN, CDHR3, CCDC78, FAM92B*) and negative loadings on EMT-specific genes (*DCN, SFRP4, TIMP3, MFAP4, CRISPLD2, SPARC*). Because these canonical ciliated markers were not available on the CosMx platform, this component is only moderately recovered in the 20-gene panel ($PC1_{52}$ vs $PC2_{20}$: $r = -0.661$; Supplementary Figure 6A), with the residual correlation driven by shared EMT markers such as *DCN*. The ciliated programme is instead represented through proxy markers (*KRT23, IGFBP5*), which retain classification capacity but with reduced specificity. However, this has limited impact on the present study, as the primary findings concern EMT-based prognostic stratification and spatial organisation, and the EMT-relevant axes of variation are strongly preserved.

Collectively, these findings demonstrate that the 20-gene subset preserves the underlying latent structure and maintains the discriminatory capacity required for cell state classification, notwithstanding the relaxed dispersion threshold adopted to ensure compatibility with the CosMx panel.

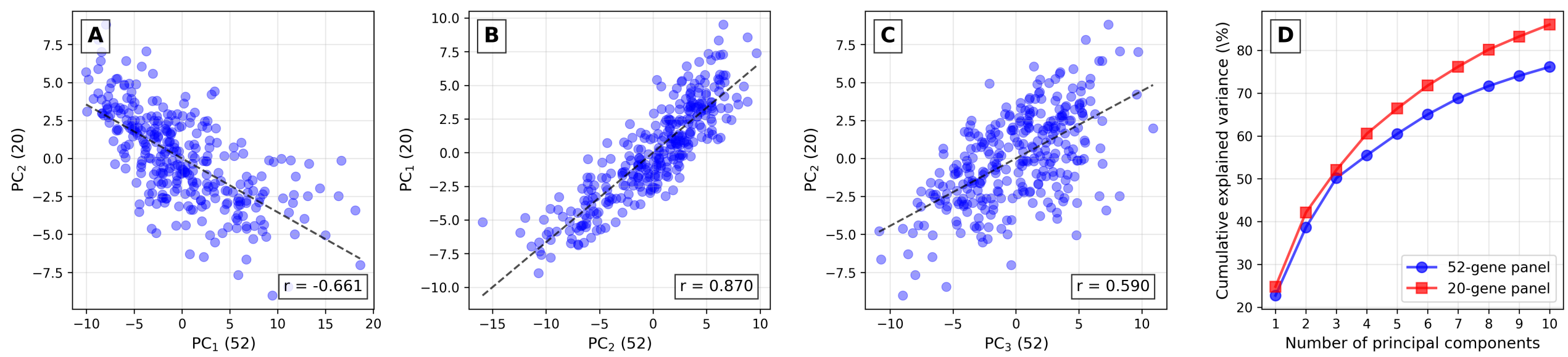

Supplementary Figure 6: PCA validation of 20-gene signature in the TCGA dataset. (**A**) PC1 from the 52-gene panel, which captures the ciliated-versus-EMT axis, shows moderate correlation with PC2 of the 20-gene panel ($r = -0.661$), reflecting partial recovery of this variation through shared EMT markers. (**B–C**) Scatter plots show strong correlations between subsequent matched principal components from the 52-gene and 20-gene panels ($PC2_{52}$ vs $PC1_{20}$: $r = 0.870$; $PC3_{52}$ vs $PC2_{20}$: $r = 0.590$). (**D**) Cumulative variance explained demonstrates efficient information retention in the reduced gene set.

Furthermore, the EMT-based risk stratification was validated in *non-spatial* bulk-expression cohorts corresponding to those analysed in [2]: TCGA and AOCS, together with six additional microarray datasets from CuratedOvarianData [3]. For each cohort, EMT proportions were computed by CIBERSORTx deconvolution using the 20-gene signature matrix developed in this study, then dichotomised at the within-cohort median (EMT-high vs EMT-low). Kaplan–Meier estimates were pooled across cohorts (total $n = 1657$; Supplementary Figure S7). EMT-high showed significantly worse overall

survival (unstratified log-rank: $\chi^2 = 20.57$, $p = 5.76 \times 10^{-6}$), and the effect remained after controlling for between-study variability (stratified log-rank by database: $\chi^2 = 21.15$, $p = 4.24 \times 10^{-6}$). These results replicate the adverse prognostic association of EMT reported in the OxC paper while confirming that it is preserved when applying the modified 20-gene signature matrix used here.

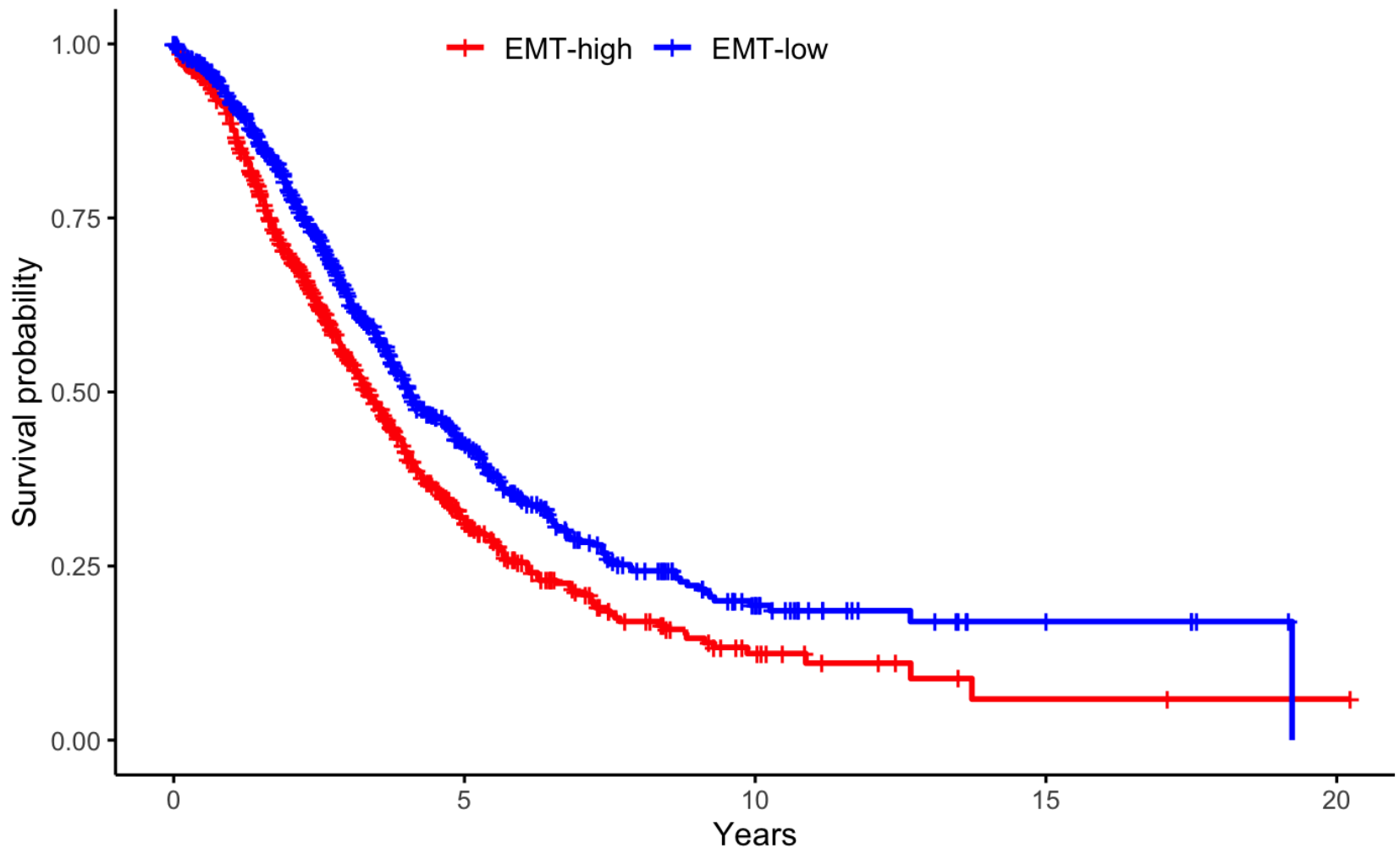

Supplementary Figure 7: Kaplan-Meier curves showing the effect of EMT scores on survival using the modified 20-gene OxC signature matrix. Patients were dichotomized into EMT-high and EMT-low by the median of EMT scores in each dataset. Each short vertical line indicates a censoring event

3 Spatial metrics - summary and statistical comparison

Summary statistics of the spatial metrics are presented in the tables below. As such, neighbourhood enrichment values are shown in Supplementary Table 1, co-localization quotient values are presented in Supplementary Table 2, while the area under the Ripley's L function curve (AUC) and degree centrality metrics are presented in Supplementary Tables 3 and 4 respectively. All statistical analyses presented here were performed using GraphPad Prism version 10.5.0 (GraphPad Inc., La Jolla, CA, USA).

Supplementary Table 1: Summary of neighbourhood enrichment z-scores for EMT cells relative to other cell types. Statistical significance was assessed using a two-tailed Wilcoxon signed-rank test against the null expectation (median = 0).

Cell type	Support (n)	Mean	SD	Median	p-value	Significance
T/NK cells	54	−5.50	5.70	−4.22	< 0.0001	****
B cells	48	−4.12	3.91	−3.58	< 0.0001	****
Mast cells	38	−1.89	1.35	−1.85	< 0.0001	****
Monocytes	54	−6.11	8.09	−4.62	< 0.0001	****
Fibroblasts	54	−14.59	13.97	−11.23	< 0.0001	****
Endothelial	48	−4.12	3.91	−3.58	< 0.0001	****

Supplementary Table 2: Summary of pairwise co-localization quotient (CLQ) values of EMT-like cells relative to major cell types. CLQ values represent observed-to-expected ratios within a $30\mu m$ radius; higher values indicate greater local co-occurrence. Statistical significance was assessed using a two-tailed Wilcoxon signed-rank test against the null expectation (median = 1).

Cell Type	Support (n)	Mean	SD	Median	p-value	Significance
T/NK cells	49	16.83	10.83	16.45	< 0.0001	****
B cells	25	8.30	10.93	5.17	< 0.0001	****
Mast cells	3	15.83	7.80	13.01	0.2500	ns
Monocytes	52	20.55	12.00	17.37	< 0.0001	****
Fibroblasts	51	14.48	15.34	9.48	< 0.0001	****
Endothelial	53	13.54	10.88	12.61	< 0.0001	****

Supplementary Table 3: Summary statistics of Ripley's L AUC values across cell populations. Higher AUC reflects greater spatial clustering or aggregation over increasing spatial radii.

Cell Type	Mean	Median	SD
EMT	662,577	483,125	659,027
T/NK cells	224,451	142,532	230,086
B cells	140,152	52,811	214,500
Mast cells	12,372	8,096	13,928
Monocytes	349,396	282,326	234,217
Fibroblasts	722,744	549,904	632,398
Endothelial	99,623	80,129	67,824

Supplementary Table 4: Summary statistics of degree centrality values for each cell population. Higher values indicate stronger inter-cluster connectivity within the spatial graph.

Cell Type	Mean	Median	SD
EMT	0.3261	0.2717	0.2310
T/NK cells	0.3640	0.3267	0.2348
B cells	0.1227	0.0556	0.1517
Mast cells	0.0294	0.0201	0.0289
Monocytes	0.5778	0.5746	0.2165
Fibroblasts	0.4276	0.3705	0.2791
Endothelial cells	0.1969	0.1632	0.1003

For Ripley's statistics and degree centrality metrics, the statistical comparison of spatial metrics was conducted using post-hoc multiple comparisons following the Kruskal–Wallis test, with Dunn's correction for multiple testing. Results are summarized in the tables below, with a significance threshold of $\alpha = 0.05$. Statistically significant differences are highlighted accordingly ($p \geq 0.05 =$ ns, $p < 0.05 = *$, $p < 0.01 = **$, $p < 0.001 = ***$ and $p < 0.0001 = ****$).

Supplementary Table 5: Statistical comparison - Ripley's L AUC.

Comparison	Mean Rank Diff.	Adj. p-value	Significance
EMT vs. T/NK	63.70	0.0514	ns
EMT vs. B cells	124.8	< 0.0001	****
EMT vs. Mast cells	211.7	< 0.0001	****
EMT vs. Monocytes	11.77	> 0.9999	ns
EMT vs. Fibroblasts	−6.676	> 0.9999	ns
EMT vs. Endothelial	105.7	< 0.0001	****
T/NK vs. B cells	61.10	0.0769	ns
T/NK vs. Mast cells	148.0	< 0.0001	****
T/NK vs. Monocytes	−51.94	0.2838	ns
T/NK vs. Fibroblasts	−70.38	0.0172	*
T/NK vs. Endothelial	42.02	0.9595	ns
B cells vs. Mast cells	86.87	0.0008	***
B cells vs. Monocytes	−113.0	< 0.0001	****
B cells vs. Fibroblasts	−131.5	< 0.0001	****
B cells vs. Endothelial	−19.08	> 0.9999	ns
Mast cells vs. Monocytes	−199.9	< 0.0001	****
Mast cells vs. Fibroblasts	−218.4	< 0.0001	****
Mast cells vs. Endothelial	−106.0	< 0.0001	****
Monocytes vs. Fibroblasts	−18.44	> 0.9999	ns
Monocytes vs. Endothelial	93.95	0.0002	***
Fibroblasts vs. Endothelial	112.4	< 0.0001	****

Supplementary Table 6: Statistical comparison - Degree centrality.

Comparison	Mean Rank Diff.	Adj. p-value	Significance
EMT vs. T/NK	−14.17	> 0.9999	ns
EMT vs. B cells	97.51	< 0.0001	****
EMT vs. Mast cells	150.8	< 0.0001	****
EMT vs. Monocytes	−84.31	0.0004	***
EMT vs. Fibroblasts	−32.04	> 0.9999	ns
EMT vs. Endothelial	44.89	0.4919	ns
T/NK vs. B cells	111.7	< 0.0001	****
T/NK vs. Mast cells	165.0	< 0.0001	****
T/NK vs. Monocytes	−70.15	0.0083	**
T/NK vs. Fibroblasts	−17.87	> 0.9999	ns
T/NK vs. Endothelial	59.06	0.0602	ns
B cells vs. Mast cells	53.33	0.3572	ns
B cells vs. Monocytes	−181.8	< 0.0001	****
B cells vs. Fibroblasts	−129.5	< 0.0001	****
B cells vs. Endothelial	−52.62	0.2090	ns
Mast cells vs. Monocytes	−235.2	< 0.0001	****
Mast cells vs. Fibroblasts	−182.9	< 0.0001	****
Mast cells vs. Endothelial	−105.9	< 0.0001	****
Monocytes vs. Fibroblasts	52.28	0.1743	ns
Monocytes vs. Endothelial	129.2	< 0.0001	****
Fibroblasts vs. Endothelial	76.93	0.0022	**

Tables 7-10 present the statistical comparison across spatial metrics for the EMT-low/high stratification.

Supplementary Table 7: Dunn's multiple comparisons test for neighbourhood enrichment values between EMT-High and EMT-Low groups.

Cell type	Mean rank diff.	Adjusted p-value	Significance
T/NK Cells	73.52	0.0119	*
B Cells	57.50	0.1353	ns
Mast Cells	38.01	> 0.9999	ns
Monocytes	99.33	0.0002	***
Fibroblasts	119.10	< 0.0001	****
Endothelial	74.89	0.0098	**

Supplementary Table 8: Dunn's multiple comparisons test for CLQ values between EMT-High and EMT-Low groups.

Cell type	Mean rank diff.	Adjusted p-value	Significance
T/NK Cells	26.24	> 0.9999	n.s.
B Cells	26.79	> 0.9999	n.s.
Mast Cells	131.70	0.5526	n.s.
Monocytes	36.52	0.3117	n.s.
Fibroblasts	33.00	0.4936	n.s.
Endothelial	25.65	> 0.9999	n.s.

Supplementary Table 9: Dunn's multiple comparisons test for Ripley's L statistic AUC between EMT-High and EMT-Low groups.

Cell type	Mean rank diff.	Adjusted p-value	Significance
EMT	-120.10	0.0001	***
T/NK Cells	17.41	> 0.9999	n.s.
B Cells	18.92	> 0.9999	n.s.
Mast Cells	-2.72	> 0.9999	n.s.
Monocytes	0.00	> 0.9999	n.s.
Fibroblasts	-84.78	0.0173	*
Endothelial	-7.48	> 0.9999	n.s.

Supplementary Table 10: Dunn's multiple comparisons test for degree centrality values between EMT-High and EMT-Low groups.

Cell type	Mean rank diff.	Adjusted p-value	Significance
EMT	-121.50	0.0001	***
T/NK Cells	45.04	0.7549	n.s.
B Cells	20.75	> 0.9999	n.s.
Mast Cells	-0.50	> 0.9999	n.s.
Monocytes	16.74	> 0.9999	n.s.
Fibroblasts	-63.00	0.1715	n.s.
Endothelial	0.56	> 0.9999	n.s.

3.1 Decomposition of EMT boundary connectivity

To characterise which cell populations contribute to EMT degree centrality, we examined the cell-type composition of non-EMT cells at EMT cluster boundaries. For each field of view, the spatial connectivity graph at the $30\mu m$ radius was used to identify all non-EMT cells directly connected to at least one EMT cell. The proportion of these boundary neighbours belonging to each cell type (observed proportion) was then compared to that cell type's share of the overall non-EMT tissue composition (expected proportion) to obtain an over-representation ratio. Values greater than one indicate that a cell type is found at EMT boundaries more frequently than its overall tissue abundance would predict; values less than one indicate under-representation. This measure is distinct from the permutation-based neighbourhood enrichment z-scores reported in Supplementary Table 1, which test whether the total number of heterotypic edges between two cell types exceeds random expectation across the entire field of view. Results were aggregated as medians across all FOVs containing at least five EMT cells. The results are presented in Supplementary Table 11.

Supplementary Table 11: EMT heterotypic neighbour composition at $30\mu m$. Observed proportion reflects each cell type's share of non-EMT neighbours at EMT cluster boundaries; expected proportion reflects that cell type's share of the overall non-EMT tissue composition. O/E ratio > 1 indicates over-representation at EMT boundaries. Values are medians across 87 FOVs.

Compartment	Cell type	Observed prop.	Expected prop.	O/E ratio
Malignant subtypes (total: 67.4%)	Ciliated	0.137	0.085	1.05
	C10	0.103	0.075	1.19
	C4	0.097	0.077	1.02
	C3	0.069	0.052	1.15
Stromal / Immune (total: 32.6%)	Monocyte	0.107	0.096	1.27
	Fibroblast	0.079	0.164	0.66
	T/NK cell	0.032	0.036	0.94
	Endothelial	0.015	0.025	0.72
	B cell	0.001	0.006	0.21
	Mast cell	< 0.001	0.002	0.19

4 Survival modeling - Multiple imputation strategy

Missing data in the baseline clinical variables were handled using Multiple Imputation by Chained Equations (MICE), resulting in $m = 20$ imputed datasets. Categorical variables were encoded numerically for imputation: age ($\leq 65 = 0$, $> 65 = 1$), stage (III = 3, IV = 4), neoadjuvant chemotherapy status (Untreated = 0, Treated = 1), and BRCA mutation status (Benign = 0, Pathogenic = 1). Tumor mutational burden (TMB) was log-transformed ($\log(\text{TMB} + 0.01)$) prior to imputation. Iterative imputation was performed using Bayesian Ridge regression with default settings, except for the maximum number of iterations, which was set to 100. Imputed categorical values were clipped to valid ranges and rounded to nearest integers to maintain categorical integrity.

To validate the quality of multiple imputation, we assessed whether imputed values preserved the distributional characteristics of observed data. For each variable with missing data (stage, BRCA status, and TMB), we compared the distribution of observed complete cases against imputed values across all 20 imputed datasets using the Mann-Whitney U test. For continuous variables (TMB), we additionally computed Cohen's d effect size to quantify the magnitude of any distributional shift. All variables demonstrated acceptable imputation quality. The Mann-Whitney U test confirmed that imputed distributions were statistically indistinguishable from observed data: stage ($p = 0.897$), BRCA status ($p = 0.834$), and TMB ($p = 0.436$). For TMB, Cohen's $d = -0.029$ indicated a negligible effect size. These results confirm that the MICE procedure successfully preserved the underlying data structure without introducing systematic bias.

5 Cox proportional hazards assumption check

The proportional hazards (PH) assumption, which requires that hazard ratios remain constant over time, was formally tested for all covariates in the multivariable Cox regression models. Schoenfeld residual tests were performed independently on each of the 20 multiply-imputed datasets, and results were pooled to provide robust estimates of PH validity across clinical, molecular, and spatial features. As shown in Supplementary Table 12, no systematic violations were detected, with all covariates demonstrating mean p-values greater than 0.05, confirming the appropriateness of the Cox proportional hazards framework for our survival analysis.

Supplementary Table 12: Proportional hazards assumption test results - Schoenfeld residuals.

Covariate	Mean p-value	SD p-value	Prop. Non-Sig.	Result
Clinical and Genomic Features				
Age (> 65 vs ≤ 65)	0.395	0.180	1.00	Pass
Stage (IV vs III)	0.772	0.163	1.00	Pass
Neoadjuvant Chemotherapy	0.617	0.201	1.00	Pass
BRCA Mutation	0.392	0.327	0.70	Pass
Tumor Mutational Burden (log)	0.395	0.274	0.90	Pass
Molecular Features				
EMT Cell Proportion	0.576	0.236	1.00	Pass
Spatial Features				
Monocyte Centrality	0.608	0.265	1.00	Pass
Monocyte Clustering (Ripley)	0.637	0.265	1.00	Pass
Mast Cell Centrality	0.541	0.219	1.00	Pass
Mast Cell Clustering (Ripley)	0.370	0.174	1.00	Pass
TNK Cell Clustering (Ripley)	0.243	0.193	0.90	Pass
EMT-Monocyte Co-localization	0.142	0.112	0.85	Pass
EMT-Endothelial Enrichment	0.384	0.205	1.00	Pass
EMT-Mast Cell Enrichment	0.405	0.176	1.00	Pass

6 Survival modeling - Individual spatial feature performance

To assess the robustness of individual spatial features on survival outcomes, each feature was added to the EMT-augmented baseline Cox model and independently evaluated across $m = 20$ imputed datasets. Model performance was summarized by the pooled C-index and mean hazard ratio (HR). The analysis further quantified the *selection stability*, which is defined as the proportion of imputations in which the penalized Cox model assigned a nonzero coefficient to the feature. High stability values (close to 1.00) indicate that a feature was consistently retained across imputations, reflecting a reproducible prognostic signal, whereas low stability suggests greater sensitivity to dataset variability.

Supplementary Table 13: Performance of individual spatial features added to the EMT-augmented Cox model. Values are averaged across $m = 20$ imputations.

Spatial Feature	C-index (mean ± SD)	Selection Stability	Mean Coefficient	Mean HR
Monocyte Centrality	0.771 ± 0.013	1.00	−0.169	0.845
Monocyte Clustering (Ripley)	0.761 ± 0.012	1.00	−0.173	0.841
Mast Cell Centrality	0.743 ± 0.013	1.00	−0.130	0.878
T/NK Cell Clustering (Ripley)	0.735 ± 0.015	1.00	−0.126	0.882
Mast Cell Clustering (Ripley)	0.731 ± 0.013	1.00	−0.145	0.865
EMT–Monocyte CLQ	0.718 ± 0.013	1.00	−0.009	0.991
EMT–Mast Cell Enrichment	0.715 ± 0.011	1.00	−0.009	0.991
EMT–Endothelial Enrichment	0.714 ± 0.010	1.00	−0.068	0.934
EMT–T/NK Cell CLQ	0.713 ± 0.015	0.05	0.000	1.000
EMT–T/NK Cell Enrichment	0.713 ± 0.015	0.00	0.000	1.000
Fibroblast Clustering (Ripley)	0.713 ± 0.015	0.00	0.000	1.000
EMT–Endothelial CLQ	0.713 ± 0.015	0.00	0.000	1.000
EMT–Fibroblast CLQ	0.713 ± 0.015	0.00	0.000	1.000
EMT–Fibroblast Enrichment	0.712 ± 0.016	1.00	−0.061	0.941
EMT–Monocyte Enrichment	0.711 ± 0.013	1.00	−0.026	0.974
EMT–B Cell Enrichment	0.711 ± 0.016	0.90	0.006	1.006
B Cell Clustering (Ripley)	0.709 ± 0.015	1.00	−0.033	0.967
EMT Clustering (Ripley)	0.708 ± 0.009	1.00	0.063	1.065
Endothelial Centrality	0.704 ± 0.017	1.00	−0.035	0.966
Endothelial Clustering (Ripley)	0.704 ± 0.014	1.00	−0.050	0.952

Supplementary References

1. Steen, C. B., Liu, C. L., Alizadeh, A. A. & Newman, A. M. Profiling cell type abundance and expression in bulk tissues with CIBERSORTx. *Stem Cell Transcriptional Networks: Methods and Protocols,* 135–157 (2020).
2. Hu, Z. *et al.* The repertoire of serous ovarian cancer non-genetic heterogeneity revealed by single-cell sequencing of normal fallopian tube epithelial cells. *Cancer cell* **37,** 226–242 (2020).
3. Ganzfried, B. F. *et al.* curatedOvarianData: clinically annotated data for the ovarian cancer transcriptome. *Database* **2013** (2013).
