# Supplementary material for: Understanding the spatial determinants of the Oxford Classic prognostic signature for high-grade serous ovarian cancer
Source: Commun Med (Lond). 2026 Jun 6;6:484. doi: 10.1038/s43856-026-01708-1 (PMC13574789; doi:10.1038/s43856-026-01708-1)
Supplement: Supplementary file 2 [file 43856_2026_1708_MOESM2_ESM.docx]

**Description of Additional Supplementary Files**

File Name- Supplementary Data 1

Description: Source data for Figures 3-7
